# Supplementary material for: Genetic diversity and SNP’s from the chloroplast coding regions of virus-infected cassava
Source: PeerJ. 2020 Mar 2;8:e8632. doi: 10.7717/peerj.8632 (PMC7058106; doi:10.7717/peerj.8632)
Supplement: Supplemental Information 1 [file peerj-08-8632-s001.pdf]

	T03	T04	T05	T06	T07	T08	T09	T10	T11	T12	K01	K02	K03	K04	K05	K07	K10	K11	K12	K13	K14	K15	M4	M5	M8	M10	M11	M16	M17	M20	M23	DRJL030	FLA444	Unk_T...	Tanz_...	TMS I5...	Unk_N...	Moz_M...	Tanz_...
T03		1	4	3	1	6	0	2	3	1	17	28	27	28	18	14	14	23	20	18	4	29	9	10	8	10	16	15	17	3	18	23	28	28	23	23	28	50	70
T04	1		3	2	2	7	1	3	4	2	16	27	26	27	19	13	13	22	19	19	5	30	10	11	9	11	15	14	16	4	17	22	27	27	22	22	27	51	69
T05	4	3		3	3	8	4	2	2	3	13	24	23	24	16	12	12	19	16	16	3	29	9	8	8	10	12	11	13	3	14	19	24	24	19	19	24	50	66
T06	3	2	3		2	7	3	3	4	2	14	25	24	25	19	13	13	20	17	19	5	30	10	11	9	11	15	12	14	4	15	20	25	25	20	20	25	51	67
T07	1	2	3	2		5	1	1	2	0	16	27	26	27	17	13	13	22	19	17	3	28	8	9	7	9	15	14	16	2	17	22	27	27	22	22	27	49	69
T08	6	7	8	7	5		6	6	7	5	21	32	31	32	22	16	16	27	24	20	8	31	13	14	12	14	20	19	21	7	22	27	32	32	27	27	32	54	74
T09	0	1	4	3	1	6		2	3	1	17	28	27	28	18	14	14	23	20	18	4	29	9	10	8	10	16	15	17	3	18	23	28	28	23	23	28	50	70
T10	2	3	2	3	1	6	2		2	1	15	26	25	26	16	14	14	21	18	16	3	29	9	8	8	10	14	13	15	3	16	21	26	26	21	21	26	50	68
T11	3	4	2	4	2	7	3	2		2	15	26	25	26	16	13	13	21	18	16	1	28	8	8	7	9	14	13	15	2	16	21	26	26	21	21	26	49	68
T12	1	2	3	2	0	5	1	1	2		16	27	26	27	17	13	13	22	19	17	3	28	8	9	7	9	15	14	16	2	17	22	27	27	22	22	27	49	69
K01	17	16	13	14	16	21	17	15	15	16		11	10	11	9	13	13	16	13	27	14	30	10	7	9	11	9	10	10	14	13	16	17	11	16	16	11	57	59
K02	28	27	24	25	27	32	28	26	26	27	11		1	0	20	24	24	5	14	38	25	41	19	18	20	20	12	13	13	25	10	5	6	0	5	5	0	68	48
K03	27	26	23	24	26	31	27	25	25	26	10	1		1	19	23	23	6	13	37	24	40	18	17	19	19	11	12	12	24	9	6	7	1	6	6	1	67	49
K04	28	27	24	25	27	32	28	26	26	27	11	0	1		20	24	24	5	14	38	25	41	19	18	20	20	12	13	13	25	10	5	6	0	5	5	0	68	48
K05	18	19	16	19	17	22	18	16	16	17	9	20	19	20		16	16	25	18	22	17	29	11	8	10	12	14	19	19	15	22	25	26	20	25	25	20	58	68
K07	14	13	12	13	13	16	14	14	13	13	13	24	23	24	16		1	29	26	16	14	17	7	8	6	8	14	21	23	11	24	29	30	24	29	29	24	54	72
K10	14	13	12	13	13	16	14	14	13	13	13	24	23	24	16	1		29	26	17	14	18	7	8	6	8	14	21	23	11	24	29	30	24	29	29	24	54	72
K11	23	22	19	20	22	27	23	21	21	22	16	5	6	5	25	29	29		9	33	20	46	24	23	25	25	17	8	8	20	5	0	5	5	0	0	5	67	47
K12	20	19	16	17	19	24	20	18	18	19	13	14	13	14	18	26	26	9		30	17	43	21	20	22	22	16	7	7	17	6	9	14	14	9	9	14	64	56
K13	18	19	16	19	17	20	18	16	16	17	27	38	37	38	22	16	17	33	30		17	23	21	20	20	22	28	25	27	15	28	33	38	38	33	33	38	62	80
K14	4	5	3	5	3	8	4	3	1	3	14	25	24	25	17	14	14	20	17	17		29	9	9	8	10	15	12	14	3	15	20	25	25	20	20	25	50	67
K15	29	30	29	30	28	31	29	29	28	28	30	41	40	41	29	17	18	46	43	23	29		22	23	21	23	31	38	40	26	41	46	47	41	46	46	41	69	89
M4	9	10	9	10	8	13	9	9	8	8	10	19	18	19	11	7	7	24	21	21	9	22		3	1	1	9	16	18	6	19	24	25	19	24	24	19	49	67
M5	10	11	8	11	9	14	10	8	8	9	7	18	17	18	8	8	8	23	20	20	9	23	3		2	4	8	15	17	7	18	23	24	18	23	23	18	50	66
M8	8	9	8	9	7	12	8	8	7	7	9	20	19	20	10	6	6	25	22	20	8	21	1	2		2	10	17	19	5	20	25	26	20	25	25	20	48	68
M10	10	11	10	11	9	14	10	10	9	9	11	20	19	20	12	8	8	25	22	22	10	23	1	4	2		10	17	19	7	20	25	26	20	25	25	20	50	68
M11	16	15	12	15	15	20	16	14	14	15	9	12	11	12	14	14	14	17	16	28	15	31	9	8	10	10		9	11	15	12	17	18	12	17	17	12	58	60
M16	15	14	11	12	14	19	15	13	13	14	10	13	12	13	19	21	21	8	7	25	12	38	16	15	17	17	9		2	12	3	8	13	13	8	8	13	59	55
M17	17	16	13	14	16	21	17	15	15	16	10	13	12	13	19	23	23	8	7	27	14	40	18	17	19	19	11	2		14	5	8	13	13	8	8	13	61	55
M20	3	4	3	4	2	7	3	3	2	2	14	25	24	25	15	11	11	20	17	15	3	26	6	7	5	7	15	12	14		15	20	25	25	20	20	25	47	67
M23	18	17	14	15	17	22	18	16	16	17	13	10	9	10	22	24	24	5	6	28	15	41	19	18	20	20	12	3	5	15		5	10	10	5	5	10	62	52
DRJL030	23	22	19	20	22	27	23	21	21	22	16	5	6	5	25	29	29	0	9	33	20	46	24	23	25	25	17	8	8	20	5		5	5	0	0	5	67	47
FLA444	28	27	24	25	27	32	28	26	26	27	17	6	7	6	26	30	30	5	14	38	25	47	25	24	26	26	18	13	13	25	10	5		6	5	5	6	68	48
Unk_TME3	28	27	24	25	27	32	28	26	26	27	11	0	1	0	20	24	24	5	14	38	25	41	19	18	20	20	12	13	13	25	10	5	6		5	5	0	68	48
Tanz_TreeCassava	23	22	19	20	22	27	23	21	21	22	16	5	6	5	25	29	29	0	9	33	20	46	24	23	25	25	17	8	8	20	5	0	5	5		0	5	67	47
TMS I50395	23	22	19	20	22	27	23	21	21	22	16	5	6	5	25	29	29	0	9	33	20	46	24	23	25	25	17	8	8	20	5	0	5	5	0		5	67	47
Unk_Namik	28	27	24	25	27	32	28	26	26	27	11	0	1	0	20	24	24	5	14	38	25	41	19	18	20	20	12	13	13	25	10	5	6	0	5	5		68	48
Moz_Mglaziovii	50	51	50	51	49	54	50	50	49	49	57	68	67	68	58	54	54	67	64	62	50	69	49	50	48	50	58	59	61	47	62	67	68	68	67	67	68		20
Tanz_Mglaziovii	70	69	66	67	69	74	70	68	68	69	59	48	49	48	68	72	72	47	56	80	67	89	67	66	68	68	60	55	55	67	52	47	48	48	47	47	48	20	
