# Supplementary material for: Genetic diversity and SNP’s from the chloroplast coding regions of virus-infected cassava
Source: PeerJ. 2020 Mar 2;8:e8632. doi: 10.7717/peerj.8632 (PMC7058106; doi:10.7717/peerj.8632)
Supplement: Supplemental Information 2 [file peerj-08-8632-s002.docx]

| **Gene** | **Product** | **Sample** | **GenBank Accession** |
| --- | --- | --- | --- |
| psbA | photosystem II protein D1 | Tanzania_Healthy | MK427095 |
| psbA | photosystem II protein D1 | Tanzania_03_Rumara | MK427096 |
| psbA | photosystem II protein D1 | Tanzania_04_Kibandameno | MK427097 |
| psbA | photosystem II protein D1 | Tanzania_05_Mkunungu | MK427098 |
| psbA | photosystem II protein D1 | Tanzania_06_Kibandameno | MK427099 |
| psbA | photosystem II protein D1 | Tanzania_07_Unknown | MK427100 |
| psbA | photosystem II protein D1 | Tanzania_08_Unknown | MK427101 |
| psbA | photosystem II protein D1 | Tanzania_09_Mkangawandu | MK427102 |
| psbA | photosystem II protein D1 | Tanzania_10_Kibembe | MK427103 |
| psbA | photosystem II protein D1 | Tanzania_11_Kibembe | MK427104 |
| psbA | photosystem II protein D1 | Tanzania_12_Mwarabu | MK427105 |
| psbA | photosystem II protein D1 | Kenya_01_Local | MK427106 |
| psbA | photosystem II protein D1 | Kenya_02_Megana | MK427107 |
| psbA | photosystem II protein D1 | Kenya_03_Tereka | MK427108 |
| psbA | photosystem II protein D1 | Kenya_04_Megana | MK427109 |
| psbA | photosystem II protein D1 | Kenya_05_Megana | MK427110 |
| psbA | photosystem II protein D1 | Kenya_07_Local | MK427111 |
| psbA | photosystem II protein D1 | Kenya_10_Local | MK427112 |
| psbA | photosystem II protein D1 | Kenya_11_Kibandameno | MK427113 |
| psbA | photosystem II protein D1 | Kenya_12_Local | MK427114 |
| psbA | photosystem II protein D1 | Kenya_13_Kibandameno | MK427115 |
| psbA | photosystem II protein D1 | Kenya_14_Kibandameno | MK427116 |
| psbA | photosystem II protein D1 | Kenya_15_Kibandameno | MK427117 |
| psbA | photosystem II protein D1 | Mozambique_4_Ezalamalithi | MK427118 |
| psbA | photosystem II protein D1 | Mozambique_5_Calamidade | MK427119 |
| psbA | photosystem II protein D1 | Mozambique_8_Calamidade | MK427120 |
| psbA | photosystem II protein D1 | Mozambique_10_Bwana | MK427121 |
| psbA | photosystem II protein D1 | Mozambique_11_Fernando | MK427122 |
| psbA | photosystem II protein D1 | Mozambique_16_Bwana | MK427123 |
| psbA | photosystem II protein D1 | Mozambique_17_Mulaleia | MK427124 |
| psbA | photosystem II protein D1 | Mozambique_20_Cadri | MK427125 |
| psbA | photosystem II protein D1 | Mozambique_23_Mulaleia | MK427126 |
| psbA | photosystem II protein D1 | Mozambique_Manihot_glaziovii | MK427127 |
| ycf4 | photosystem I assembly protein ycf4 | Tanzania_Healthy | MK427128 |
| ycf4 | photosystem I assembly protein ycf4 | Tanzania_03_Rumara | MK427129 |
| ycf4 | photosystem I assembly protein ycf4 | Tanzania_04_Kibandameno | MK427130 |
| ycf4 | photosystem I assembly protein ycf4 | Tanzania_05_Mkunungu | MK427131 |
| ycf4 | photosystem I assembly protein ycf4 | Tanzania_06_Kibandameno | MK427132 |
| ycf4 | photosystem I assembly protein ycf4 | Tanzania_07_Unknown | MK427133 |
| ycf4 | photosystem I assembly protein ycf4 | Tanzania_08_Unknown | MK427134 |
| ycf4 | photosystem I assembly protein ycf4 | Tanzania_09_Mkangawandu | MK427135 |
| ycf4 | photosystem I assembly protein ycf4 | Tanzania_10_Kibembe | MK427136 |
| ycf4 | photosystem I assembly protein ycf4 | Tanzania_11_Kibembe | MK427137 |
| ycf4 | photosystem I assembly protein ycf4 | Tanzania_12_Mwarabu | MK427138 |
| ycf4 | photosystem I assembly protein ycf4 | Kenya_01_Local | MK427139 |
| ycf4 | photosystem I assembly protein ycf4 | Kenya_02_Megana | MK427140 |
| ycf4 | photosystem I assembly protein ycf4 | Kenya_03_Tereka | MK427141 |
| ycf4 | photosystem I assembly protein ycf4 | Kenya_04_Megana | MK427142 |
| ycf4 | photosystem I assembly protein ycf4 | Kenya_05_Megana | MK427143 |
| ycf4 | photosystem I assembly protein ycf4 | Kenya_07_Local | MK427144 |
| ycf4 | photosystem I assembly protein ycf4 | Kenya_10_Local | MK427145 |
| ycf4 | photosystem I assembly protein ycf4 | Kenya_11_Kibandameno | MK427146 |
| ycf4 | photosystem I assembly protein ycf4 | Kenya_12_Local | MK427147 |
| ycf4 | photosystem I assembly protein ycf4 | Kenya_13_Kibandameno | MK427148 |
| ycf4 | photosystem I assembly protein ycf4 | Kenya_14_Kibandameno | MK427149 |
| ycf4 | photosystem I assembly protein ycf4 | Kenya_15_Kibandameno | MK427150 |
| ycf4 | photosystem I assembly protein ycf4 | Mozambique_4_Ezalamalithi | MK427151 |
| ycf4 | photosystem I assembly protein ycf4 | Mozambique_5_Calamidade | MK427152 |
| ycf4 | photosystem I assembly protein ycf4 | Mozambique_8_Calamidade | MK427153 |
| ycf4 | photosystem I assembly protein ycf4 | Mozambique_10_Bwana | MK427154 |
| ycf4 | photosystem I assembly protein ycf4 | Mozambique_11_Fernando | MK427155 |
| ycf4 | photosystem I assembly protein ycf4 | Mozambique_16_Bwana | MK427156 |
| ycf4 | photosystem I assembly protein ycf4 | Mozambique_17_Mulaleia | MK427157 |
| ycf4 | photosystem I assembly protein ycf4 | Mozambique_20_Cadri | MK427158 |
| ycf4 | photosystem I assembly protein ycf4 | Mozambique_23_Mulaleia | MK427159 |
| ycf4 | photosystem I assembly protein ycf4 | Mozambique_Manihot_glaziovii | MK427160 |
| atpA | ATP synthase CF1 alpha subunit | Tanzania_Healthy | MK427161 |
| atpA | ATP synthase CF1 alpha subunit | Tanzania_03_Rumara | MK427162 |
| atpA | ATP synthase CF1 alpha subunit | Tanzania_04_Kibandameno | MK427163 |
| atpA | ATP synthase CF1 alpha subunit | Tanzania_05_Mkunungu | MK427164 |
| atpA | ATP synthase CF1 alpha subunit | Tanzania_06_Kibandameno | MK427165 |
| atpA | ATP synthase CF1 alpha subunit | Tanzania_07_Unknown | MK427166 |
| atpA | ATP synthase CF1 alpha subunit | Tanzania_08_Unknown | MK427167 |
| atpA | ATP synthase CF1 alpha subunit | Tanzania_09_Mkangawandu | MK427168 |
| atpA | ATP synthase CF1 alpha subunit | Tanzania_10_Kibembe | MK427169 |
| atpA | ATP synthase CF1 alpha subunit | Tanzania_11_Kibembe | MK427170 |
| atpA | ATP synthase CF1 alpha subunit | Tanzania_12_Mwarabu | MK427171 |
| atpA | ATP synthase CF1 alpha subunit | Kenya_01_Local | MK427172 |
| atpA | ATP synthase CF1 alpha subunit | Kenya_02_Megana | MK427173 |
| atpA | ATP synthase CF1 alpha subunit | Kenya_03_Tereka | MK427174 |
| atpA | ATP synthase CF1 alpha subunit | Kenya_04_Megana | MK427175 |
| atpA | ATP synthase CF1 alpha subunit | Kenya_05_Megana | MK427176 |
| atpA | ATP synthase CF1 alpha subunit | Kenya_07_Local | MK427177 |
| atpA | ATP synthase CF1 alpha subunit | Kenya_10_Local | MK427178 |
| atpA | ATP synthase CF1 alpha subunit | Kenya_11_Kibandameno | MK427179 |
| atpA | ATP synthase CF1 alpha subunit | Kenya_12_Local | MK427180 |
| atpA | ATP synthase CF1 alpha subunit | Kenya_13_Kibandameno | MK427181 |
| atpA | ATP synthase CF1 alpha subunit | Kenya_14_Kibandameno | MK427182 |
| atpA | ATP synthase CF1 alpha subunit | Kenya_15_Kibandameno | MK427183 |
| atpA | ATP synthase CF1 alpha subunit | Mozambique_4_Ezalamalithi | MK427184 |
| atpA | ATP synthase CF1 alpha subunit | Mozambique_5_Calamidade | MK427185 |
| atpA | ATP synthase CF1 alpha subunit | Mozambique_8_Calamidade | MK427186 |
| atpA | ATP synthase CF1 alpha subunit | Mozambique_10_Bwana | MK427187 |
| atpA | ATP synthase CF1 alpha subunit | Mozambique_11_Fernando | MK427188 |
| atpA | ATP synthase CF1 alpha subunit | Mozambique_16_Bwana | MK427189 |
| atpA | ATP synthase CF1 alpha subunit | Mozambique_17_Mulaleia | MK427190 |
| atpA | ATP synthase CF1 alpha subunit | Mozambique_20_Cadri | MK427191 |
| atpA | ATP synthase CF1 alpha subunit | Mozambique_23_Mulaleia | MK427192 |
| atpA | ATP synthase CF1 alpha subunit | Mozambique_Manihot_glaziovii | MK427193 |
| ycf3 | photosystem I assembly protein ycf3 | Tanzania_Healthy | MK427194 |
| ycf3 | photosystem I assembly protein ycf3 | Tanzania_03_Rumara | MK427195 |
| ycf3 | photosystem I assembly protein ycf3 | Tanzania_04_Kibandameno | MK427196 |
| ycf3 | photosystem I assembly protein ycf3 | Tanzania_05_Mkunungu | MK427197 |
| ycf3 | photosystem I assembly protein ycf3 | Tanzania_06_Kibandameno | MK427198 |
| ycf3 | photosystem I assembly protein ycf3 | Tanzania_07_Unknown | MK427199 |
| ycf3 | photosystem I assembly protein ycf3 | Tanzania_08_Unknown | MK427200 |
| ycf3 | photosystem I assembly protein ycf3 | Tanzania_09_Mkangawandu | MK427201 |
| ycf3 | photosystem I assembly protein ycf3 | Tanzania_10_Kibembe | MK427202 |
| ycf3 | photosystem I assembly protein ycf3 | Tanzania_11_Kibembe | MK427203 |
| ycf3 | photosystem I assembly protein ycf3 | Tanzania_12_Mwarabu | MK427204 |
| ycf3 | photosystem I assembly protein ycf3 | Kenya_01_Local | MK427205 |
| ycf3 | photosystem I assembly protein ycf3 | Kenya_02_Megana | MK427206 |
| ycf3 | photosystem I assembly protein ycf3 | Kenya_03_Tereka | MK427207 |
| ycf3 | photosystem I assembly protein ycf3 | Kenya_04_Megana | MK427208 |
| ycf3 | photosystem I assembly protein ycf3 | Kenya_05_Megana | MK427209 |
| ycf3 | photosystem I assembly protein ycf3 | Kenya_07_Local | MK427210 |
| ycf3 | photosystem I assembly protein ycf3 | Kenya_10_Local | MK427211 |
| ycf3 | photosystem I assembly protein ycf3 | Kenya_11_Kibandameno | MK427212 |
| ycf3 | photosystem I assembly protein ycf3 | Kenya_12_Local | MK427213 |
| ycf3 | photosystem I assembly protein ycf3 | Kenya_13_Kibandameno | MK427214 |
| ycf3 | photosystem I assembly protein ycf3 | Kenya_14_Kibandameno | MK427215 |
| ycf3 | photosystem I assembly protein ycf3 | Kenya_15_Kibandameno | MK427216 |
| ycf3 | photosystem I assembly protein ycf3 | Mozambique_4_Ezalamalithi | MK427217 |
| ycf3 | photosystem I assembly protein ycf3 | Mozambique_5_Calamidade | MK427218 |
| ycf3 | photosystem I assembly protein ycf3 | Mozambique_8_Calamidade | MK427219 |
| ycf3 | photosystem I assembly protein ycf3 | Mozambique_10_Bwana | MK427220 |
| ycf3 | photosystem I assembly protein ycf3 | Mozambique_11_Fernando | MK427221 |
| ycf3 | photosystem I assembly protein ycf3 | Mozambique_16_Bwana | MK427222 |
| ycf3 | photosystem I assembly protein ycf3 | Mozambique_17_Mulaleia | MK427223 |
| ycf3 | photosystem I assembly protein ycf3 | Mozambique_20_Cadri | MK427224 |
| ycf3 | photosystem I assembly protein ycf3 | Mozambique_23_Mulaleia | MK427225 |
| ycf3 | photosystem I assembly protein ycf3 | Mozambique_Manihot_glaziovii | MK427226 |
| atpF | ATP synthase CF0 subunit I | Tanzania_Healthy | MK427227 |
| atpF | ATP synthase CF0 subunit I | Tanzania_03_Rumara | MK427228 |
| atpF | ATP synthase CF0 subunit I | Tanzania_04_Kibandameno | MK427229 |
| atpF | ATP synthase CF0 subunit I | Tanzania_05_Mkunungu | MK427230 |
| atpF | ATP synthase CF0 subunit I | Tanzania_06_Kibandameno | MK427231 |
| atpF | ATP synthase CF0 subunit I | Tanzania_07_Unknown | MK427232 |
| atpF | ATP synthase CF0 subunit I | Tanzania_08_Unknown | MK427233 |
| atpF | ATP synthase CF0 subunit I | Tanzania_09_Mkangawandu | MK427234 |
| atpF | ATP synthase CF0 subunit I | Tanzania_10_Kibembe | MK427235 |
| atpF | ATP synthase CF0 subunit I | Tanzania_11_Kibembe | MK427236 |
| atpF | ATP synthase CF0 subunit I | Tanzania_12_Mwarabu | MK427237 |
| atpF | ATP synthase CF0 subunit I | Kenya_01_Local | MK427238 |
| atpF | ATP synthase CF0 subunit I | Kenya_02_Megana | MK427239 |
| atpF | ATP synthase CF0 subunit I | Kenya_03_Tereka | MK427240 |
| atpF | ATP synthase CF0 subunit I | Kenya_04_Megana | MK427241 |
| atpF | ATP synthase CF0 subunit I | Kenya_05_Megana | MK427242 |
| atpF | ATP synthase CF0 subunit I | Kenya_07_Local | MK427243 |
| atpF | ATP synthase CF0 subunit I | Kenya_10_Local | MK427244 |
| atpF | ATP synthase CF0 subunit I | Kenya_11_Kibandameno | MK427245 |
| atpF | ATP synthase CF0 subunit I | Kenya_12_Local | MK427246 |
| atpF | ATP synthase CF0 subunit I | Kenya_13_Kibandameno | MK427247 |
| atpF | ATP synthase CF0 subunit I | Kenya_14_Kibandameno | MK427248 |
| atpF | ATP synthase CF0 subunit I | Kenya_15_Kibandameno | MK427249 |
| atpF | ATP synthase CF0 subunit I | Mozambique_4_Ezalamalithi | MK427250 |
| atpF | ATP synthase CF0 subunit I | Mozambique_5_Calamidade | MK427251 |
| atpF | ATP synthase CF0 subunit I | Mozambique_8_Calamidade | MK427252 |
| atpF | ATP synthase CF0 subunit I | Mozambique_10_Bwana | MK427253 |
| atpF | ATP synthase CF0 subunit I | Mozambique_11_Fernando | MK427254 |
| atpF | ATP synthase CF0 subunit I | Mozambique_16_Bwana | MK427255 |
| atpF | ATP synthase CF0 subunit I | Mozambique_17_Mulaleia | MK427256 |
| atpF | ATP synthase CF0 subunit I | Mozambique_20_Cadri | MK427257 |
| atpF | ATP synthase CF0 subunit I | Mozambique_23_Mulaleia | MK427258 |
| atpF | ATP synthase CF0 subunit I | Mozambique_Manihot_glazioviI | MK427259 |
| atpH | ATP synthase CF0 subunit III | Tanzania_Healthy | MK427260 |
| atpH | ATP synthase CF0 subunit III | Tanzania_03_Rumara | MK427261 |
| atpH | ATP synthase CF0 subunit III | Tanzania_04_Kibandameno | MK427262 |
| atpH | ATP synthase CF0 subunit III | Tanzania_05_Mkunungu | MK427263 |
| atpH | ATP synthase CF0 subunit III | Tanzania_06_Kibandameno | MK427264 |
| atpH | ATP synthase CF0 subunit III | Tanzania_07_Unknown | MK427265 |
| atpH | ATP synthase CF0 subunit III | Tanzania_08_Unknown | MK427266 |
| atpH | ATP synthase CF0 subunit III | Tanzania_09_Mkangawandu | MK427267 |
| atpH | ATP synthase CF0 subunit III | Tanzania_10_Kibembe | MK427268 |
| atpH | ATP synthase CF0 subunit III | Tanzania_11_Kibembe | MK427269 |
| atpH | ATP synthase CF0 subunit III | Tanzania_12_Mwarabu | MK427270 |
| atpH | ATP synthase CF0 subunit III | Kenya_01_Local | MK427271 |
| atpH | ATP synthase CF0 subunit III | Kenya_02_Megana | MK427272 |
| atpH | ATP synthase CF0 subunit III | Kenya_03_Tereka | MK427273 |
| atpH | ATP synthase CF0 subunit III | Kenya_04_Megana | MK427274 |
| atpH | ATP synthase CF0 subunit III | Kenya_05_Megana | MK427275 |
| atpH | ATP synthase CF0 subunit III | Kenya_07_Local | MK427276 |
| atpH | ATP synthase CF0 subunit III | Kenya_10_Local | MK427277 |
| atpH | ATP synthase CF0 subunit III | Kenya_11_Kibandameno | MK427278 |
| atpH | ATP synthase CF0 subunit III | Kenya_12_Local | MK427279 |
| atpH | ATP synthase CF0 subunit III | Kenya_13_Kibandameno | MK427280 |
| atpH | ATP synthase CF0 subunit III | Kenya_14_Kibandameno | MK427281 |
| atpH | ATP synthase CF0 subunit III | Kenya_15_Kibandameno | MK427282 |
| atpH | ATP synthase CF0 subunit III | Mozambique_4_Ezalamalithi | MK427283 |
| atpH | ATP synthase CF0 subunit III | Mozambique_5_Calamidade | MK427284 |
| atpH | ATP synthase CF0 subunit III | Mozambique_8_Calamidade | MK427285 |
| atpH | ATP synthase CF0 subunit III | Mozambique_10_Bwana | MK427286 |
| atpH | ATP synthase CF0 subunit III | Mozambique_11_Fernando | MK427287 |
| atpH | ATP synthase CF0 subunit III | Mozambique_16_Bwana | MK427288 |
| atpH | ATP synthase CF0 subunit III | Mozambique_17_Mulaleia | MK427289 |
| atpH | ATP synthase CF0 subunit III | Mozambique_20_Cadri | MK427290 |
| atpH | ATP synthase CF0 subunit III | Mozambique_23_Mulaleia | MK427291 |
| atpH | ATP synthase CF0 subunit III | Mozambique_Manihot_glaziovii | MK427292 |
| rps2 | ribosomal protein S2 | Tanzania_Healthy | MK427293 |
| rps2 | ribosomal protein S2 | Tanzania_03_Rumara | MK427294 |
| rps2 | ribosomal protein S2 | Tanzania_04_Kibandameno | MK427295 |
| rps2 | ribosomal protein S2 | Tanzania_05_Mkunungu | MK427296 |
| rps2 | ribosomal protein S2 | Tanzania_06_Kibandameno | MK427297 |
| rps2 | ribosomal protein S2 | Tanzania_07_Unknown | MK427298 |
| rps2 | ribosomal protein S2 | Tanzania_08_Unknown | MK427299 |
| rps2 | ribosomal protein S2 | Tanzania_09_Mkangawandu | MK427300 |
| rps2 | ribosomal protein S2 | Tanzania_10_Kibembe | MK427301 |
| rps2 | ribosomal protein S2 | Tanzania_11_Kibembe | MK427302 |
| rps2 | ribosomal protein S2 | Tanzania_12_Mwarabu | MK427303 |
| rps2 | ribosomal protein S2 | Kenya_01_Local | MK427304 |
| rps2 | ribosomal protein S2 | Kenya_02_Megana | MK427305 |
| rps2 | ribosomal protein S2 | Kenya_03_Tereka | MK427306 |
| rps2 | ribosomal protein S2 | Kenya_04_Megana | MK427307 |
| rps2 | ribosomal protein S2 | Kenya_05_Megana | MK427308 |
| rps2 | ribosomal protein S2 | Kenya_07_Local | MK427309 |
| rps2 | ribosomal protein S2 | Kenya_10_Local | MK427310 |
| rps2 | ribosomal protein S2 | Kenya_11_Kibandameno | MK427311 |
| rps2 | ribosomal protein S2 | Kenya_12_Local | MK427312 |
| rps2 | ribosomal protein S2 | Kenya_13_Kibandameno | MK427313 |
| rps2 | ribosomal protein S2 | Kenya_14_Kibandameno | MK427314 |
| rps2 | ribosomal protein S2 | Kenya_15_Kibandameno | MK427315 |
| rps2 | ribosomal protein S2 | Mozambique_4_Ezalamalithi | MK427316 |
| rps2 | ribosomal protein S2 | Mozambique_5_Calamidade | MK427317 |
| rps2 | ribosomal protein S2 | Mozambique_8_Calamidade | MK427318 |
| rps2 | ribosomal protein S2 | Mozambique_10_Bwana | MK427319 |
| rps2 | ribosomal protein S2 | Mozambique_11_Fernando | MK427320 |
| rps2 | ribosomal protein S2 | Mozambique_16_Bwana | MK427321 |
| rps2 | ribosomal protein S2 | Mozambique_17_Mulaleia | MK427322 |
| rps2 | ribosomal protein S2 | Mozambique_20_Cadri | MK427323 |
| rps2 | ribosomal protein S2 | Mozambique_23_Mulaleia | MK427324 |
| rps2 | ribosomal protein S2 | Mozambique_Manihot_glaziovii | MK427325 |
| rpoc1 | RNA polymerase beta | Tanzania_Healthy | MK427326 |
| rpoc1 | RNA polymerase beta | Tanzania_03_Rumara | MK427327 |
| rpoc1 | RNA polymerase beta | Tanzania_04_Kibandameno | MK427328 |
| rpoc1 | RNA polymerase beta | Tanzania_05_Mkunungu | MK427329 |
| rpoc1 | RNA polymerase beta | Tanzania_06_Kibandameno | MK427330 |
| rpoc1 | RNA polymerase beta | Tanzania_07_Unknown | MK427331 |
| rpoc1 | RNA polymerase beta | Tanzania_08_Unknown | MK427332 |
| rpoc1 | RNA polymerase beta | Tanzania_09_Mkangawandu | MK427333 |
| rpoc1 | RNA polymerase beta | Tanzania_10_Kibembe | MK427334 |
| rpoc1 | RNA polymerase beta | Tanzania_11_Kibembe | MK427335 |
| rpoc1 | RNA polymerase beta | Tanzania_12_Mwarabu | MK427336 |
| rpoc1 | RNA polymerase beta | Kenya_01_Local | MK427337 |
| rpoc1 | RNA polymerase beta | Kenya_02_Megana | MK427338 |
| rpoc1 | RNA polymerase beta | Kenya_03_Tereka | MK427339 |
| rpoc1 | RNA polymerase beta | Kenya_04_Megana | MK427340 |
| rpoc1 | RNA polymerase beta | Kenya_05_Megana | MK427341 |
| rpoc1 | RNA polymerase beta | Kenya_07_Local | MK427342 |
| rpoc1 | RNA polymerase beta | Kenya_10_Local | MK427343 |
| rpoc1 | RNA polymerase beta | Kenya_11_Kibandameno | MK427344 |
| rpoc1 | RNA polymerase beta | Kenya_12_Local | MK427345 |
| rpoc1 | RNA polymerase beta | Kenya_13_Kibandameno | MK427346 |
| rpoc1 | RNA polymerase beta | Kenya_14_Kibandameno | MK427347 |
| rpoc1 | RNA polymerase beta | Kenya_15_Kibandameno | MK427348 |
| rpoc1 | RNA polymerase beta | Mozambique_4_Ezalamalithi | MK427349 |
| rpoc1 | RNA polymerase beta | Mozambique_5_Calamidade | MK427350 |
| rpoc1 | RNA polymerase beta | Mozambique_8_Calamidade | MK427351 |
| rpoc1 | RNA polymerase beta | Mozambique_10_Bwana | MK427352 |
| rpoc1 | RNA polymerase beta | Mozambique_11_Fernando | MK427353 |
| rpoc1 | RNA polymerase beta | Mozambique_16_Bwana | MK427354 |
| rpoc1 | RNA polymerase beta | Mozambique_17_Mulaleia | MK427355 |
| rpoc1 | RNA polymerase beta | Mozambique_20_Cadri | MK427356 |
| rpoc1 | RNA polymerase beta | Mozambique_23_Mulaleia | MK427357 |
| rpoc1 | RNA polymerase beta | Mozambique_Manihot_glaziovii | MK427358 |
| psbZ | photosystem II protein Z | Tanzania_Healthy | MK427359 |
| psbZ | photosystem II protein Z | Tanzania_03_Rumara | MK427360 |
| psbZ | photosystem II protein Z | Tanzania_04_Kibandameno | MK427361 |
| psbZ | photosystem II protein Z | Tanzania_05_Mkunungu | MK427362 |
| psbZ | photosystem II protein Z | Tanzania_06_Kibandameno | MK427363 |
| psbZ | photosystem II protein Z | Tanzania_07_Unknown | MK427364 |
| psbZ | photosystem II protein Z | Tanzania_08_Unknown | MK427365 |
| psbZ | photosystem II protein Z | Tanzania_09_Mkangawandu | MK427366 |
| psbZ | photosystem II protein Z | Tanzania_10_Kibembe | MK427367 |
| psbZ | photosystem II protein Z | Tanzania_11_Kibembe | MK427368 |
| psbZ | photosystem II protein Z | Tanzania_12_Mwarabu | MK427369 |
| psbZ | photosystem II protein Z | Kenya_01_Local | MK427370 |
| psbZ | photosystem II protein Z | Kenya_02_Megana | MK427371 |
| psbZ | photosystem II protein Z | Kenya_03_Tereka | MK427372 |
| psbZ | photosystem II protein Z | Kenya_04_Megana | MK427373 |
| psbZ | photosystem II protein Z | Kenya_05_Megana | MK427374 |
| psbZ | photosystem II protein Z | Kenya_07_Local | MK427375 |
| psbZ | photosystem II protein Z | Kenya_10_Local | MK427376 |
| psbZ | photosystem II protein Z | Kenya_11_Kibandameno | MK427377 |
| psbZ | photosystem II protein Z | Kenya_12_Local | MK427378 |
| psbZ | photosystem II protein Z | Kenya_13_Kibandameno | MK427379 |
| psbZ | photosystem II protein Z | Kenya_14_Kibandameno | MK427380 |
| psbZ | photosystem II protein Z | Kenya_15_Kibandameno | MK427381 |
| psbZ | photosystem II protein Z | Mozambique_4_Ezalamalithi | MK427382 |
| psbZ | photosystem II protein Z | Mozambique_5_Calamidade | MK427383 |
| psbZ | photosystem II protein Z | Mozambique_8_Calamidade | MK427384 |
| psbZ | photosystem II protein Z | Mozambique_10_Bwana | MK427385 |
| psbZ | photosystem II protein Z | Mozambique_11_Fernando | MK427386 |
| psbZ | photosystem II protein Z | Mozambique_16_Bwana | MK427387 |
| psbZ | photosystem II protein Z | Mozambique_17_Mulaleia | MK427388 |
| psbZ | photosystem II protein Z | Mozambique_20_Cadri | MK427389 |
| psbZ | photosystem II protein Z | Mozambique_23_Mulaleia | MK427390 |
| psbZ | photosystem II protein Z | Mozambique_Manihot_glaziovii | MK427391 |
| psbD | photosystem II protein D2 | Tanzania_Healthy | MK427392 |
| psbD | photosystem II protein D2 | Tanzania_03_Rumara | MK427393 |
| psbD | photosystem II protein D2 | Tanzania_04_Kibandameno | MK427394 |
| psbD | photosystem II protein D2 | Tanzania_05_Mkunungu | MK427395 |
| psbD | photosystem II protein D2 | Tanzania_06_Kibandameno | MK427396 |
| psbD | photosystem II protein D2 | Tanzania_07_Unknown | MK427397 |
| psbD | photosystem II protein D2 | Tanzania_08_Unknown | MK427398 |
| psbD | photosystem II protein D2 | Tanzania_09_Mkangawandu | MK427399 |
| psbD | photosystem II protein D2 | Tanzania_10_Kibembe | MK427400 |
| psbD | photosystem II protein D2 | Tanzania_11_Kibembe | MK427401 |
| psbD | photosystem II protein D2 | Tanzania_12_Mwarabu | MK427402 |
| psbD | photosystem II protein D2 | Kenya_01_Local | MK427403 |
| psbD | photosystem II protein D2 | Kenya_02_Megana | MK427404 |
| psbD | photosystem II protein D2 | Kenya_03_Tereka | MK427405 |
| psbD | photosystem II protein D2 | Kenya_04_Megana | MK427406 |
| psbD | photosystem II protein D2 | Kenya_05_Megana | MK427407 |
| psbD | photosystem II protein D2 | Kenya_07_Local | MK427408 |
| psbD | photosystem II protein D2 | Kenya_10_Local | MK427409 |
| psbD | photosystem II protein D2 | Kenya_11_Kibandameno | MK427410 |
| psbD | photosystem II protein D2 | Kenya_12_Local | MK427411 |
| psbD | photosystem II protein D2 | Kenya_13_Kibandameno | MK427412 |
| psbD | photosystem II protein D2 | Kenya_14_Kibandameno | MK427413 |
| psbD | photosystem II protein D2 | Kenya_15_Kibandameno | MK427414 |
| psbD | photosystem II protein D2 | Mozambique_4_Ezalamalithi | MK427415 |
| psbD | photosystem II protein D2 | Mozambique_5_Calamidade | MK427416 |
| psbD | photosystem II protein D2 | Mozambique_8_Calamidade | MK427417 |
| psbD | photosystem II protein D2 | Mozambique_10_Bwana | MK427418 |
| psbD | photosystem II protein D2 | Mozambique_11_Fernando | MK427419 |
| psbD | photosystem II protein D2 | Mozambique_16_Bwana | MK427420 |
| psbD | photosystem II protein D2 | Mozambique_17_Mulaleia | MK427421 |
| psbD | photosystem II protein D2 | Mozambique_20_Cadri | MK427422 |
| psbD | photosystem II protein D2 | Mozambique_23_Mulaleia | MK427423 |
| psbD | photosystem II protein D2 | Mozambique_Manihot_glaziovii | MK427424 |
| psbC | photosystem II CP43 chlorophyll apoprotein | Tanzania_Healthy | MK427425 |
| psbC | photosystem II CP43 chlorophyll apoprotein | Tanzania_03_Rumara | MK427426 |
| psbC | photosystem II CP43 chlorophyll apoprotein | Tanzania_04_Kibandameno | MK427427 |
| psbC | photosystem II CP43 chlorophyll apoprotein | Tanzania_05_Mkunungu | MK427428 |
| psbC | photosystem II CP43 chlorophyll apoprotein | Tanzania_06_Kibandameno | MK427429 |
| psbC | photosystem II CP43 chlorophyll apoprotein | Tanzania_07_Unknown | MK427430 |
| psbC | photosystem II CP43 chlorophyll apoprotein | Tanzania_08_Unknown | MK427431 |
| psbC | photosystem II CP43 chlorophyll apoprotein | Tanzania_09_Mkangawandu | MK427432 |
| psbC | photosystem II CP43 chlorophyll apoprotein | Tanzania_10_Kibembe | MK427433 |
| psbC | photosystem II CP43 chlorophyll apoprotein | Tanzania_11_Kibembe | MK427434 |
| psbC | photosystem II CP43 chlorophyll apoprotein | Tanzania_12_Mwarabu | MK427435 |
| psbC | photosystem II CP43 chlorophyll apoprotein | Kenya_01_Local | MK427436 |
| psbC | photosystem II CP43 chlorophyll apoprotein | Kenya_02_Megana | MK427437 |
| psbC | photosystem II CP43 chlorophyll apoprotein | Kenya_03_Tereka | MK427438 |
| psbC | photosystem II CP43 chlorophyll apoprotein | Kenya_04_Megana | MK427439 |
| psbC | photosystem II CP43 chlorophyll apoprotein | Kenya_05_Megana | MK427440 |
| psbC | photosystem II CP43 chlorophyll apoprotein | Kenya_07_Local | MK427441 |
| psbC | photosystem II CP43 chlorophyll apoprotein | Kenya_10_Local | MK427442 |
| psbC | photosystem II CP43 chlorophyll apoprotein | Kenya_11_Kibandameno | MK427443 |
| psbC | photosystem II CP43 chlorophyll apoprotein | Kenya_12_Local | MK427444 |
| psbC | photosystem II CP43 chlorophyll apoprotein | Kenya_13_Kibandameno | MK427445 |
| psbC | photosystem II CP43 chlorophyll apoprotein | Kenya_14_Kibandameno | MK427446 |
| psbC | photosystem II CP43 chlorophyll apoprotein | Kenya_15_Kibandameno | MK427447 |
| psbC | photosystem II CP43 chlorophyll apoprotein | Mozambique_4_Ezalamalithi | MK427448 |
| psbC | photosystem II CP43 chlorophyll apoprotein | Mozambique_5_Calamidade | MK427449 |
| psbC | photosystem II CP43 chlorophyll apoprotein | Mozambique_8_Calamidade | MK427450 |
| psbC | photosystem II CP43 chlorophyll apoprotein | Mozambique_10_Bwana | MK427451 |
| psbC | photosystem II CP43 chlorophyll apoprotein | Mozambique_11_Fernando | MK427452 |
| psbC | photosystem II CP43 chlorophyll apoprotein | Mozambique_16_Bwana | MK427453 |
| psbC | photosystem II CP43 chlorophyll apoprotein | Mozambique_17_Mulaleia | MK427454 |
| psbC | photosystem II CP43 chlorophyll apoprotein | Mozambique_20_Cadri | MK427455 |
| psbC | photosystem II CP43 chlorophyll apoprotein | Mozambique_23_Mulaleia | MK427456 |
| psbC | photosystem II CP43 chlorophyll apoprotein | Mozambique_Manihot_glaziovii | MK427457 |
| psbZ | photosystem II protein Z | Tanzania_Healthy | MK427458 |
| psbZ | photosystem II protein Z | Tanzania_03_Rumara | MK427459 |
| psbZ | photosystem II protein Z | Tanzania_04_Kibandameno | MK427460 |
| psbZ | photosystem II protein Z | Tanzania_05_Mkunungu | MK427461 |
| psbZ | photosystem II protein Z | Tanzania_06_Kibandameno | MK427462 |
| psbZ | photosystem II protein Z | Tanzania_07_Unknown | MK427463 |
| psbZ | photosystem II protein Z | Tanzania_08_Unknown | MK427464 |
| psbZ | photosystem II protein Z | Tanzania_09_Mkangawandu | MK427465 |
| psbZ | photosystem II protein Z | Tanzania_10_Kibembe | MK427466 |
| psbZ | photosystem II protein Z | Tanzania_11_Kibembe | MK427467 |
| psbZ | photosystem II protein Z | Tanzania_12_Mwarabu | MK427468 |
| psbZ | photosystem II protein Z | Kenya_01_Local | MK427469 |
| psbZ | photosystem II protein Z | Kenya_02_Megana | MK427470 |
| psbZ | photosystem II protein Z | Kenya_03_Tereka | MK427471 |
| psbZ | photosystem II protein Z | Kenya_04_Megana | MK427472 |
| psbZ | photosystem II protein Z | Kenya_05_Megana | MK427473 |
| psbZ | photosystem II protein Z | Kenya_07_Local | MK427474 |
| psbZ | photosystem II protein Z | Kenya_10_Local ` | MK427475 |
| psbZ | photosystem II protein Z | Kenya_11_Kibandameno | MK427476 |
| psbZ | photosystem II protein Z | Kenya_12_Local | MK427477 |
| psbZ | photosystem II protein Z | Kenya_13_Kibandameno | MK427478 |
| psbZ | photosystem II protein Z | Kenya_14_Kibandameno | MK427479 |
| psbZ | photosystem II protein Z | Kenya_15_Kibandameno | MK427480 |
| psbZ | photosystem II protein Z | Mozambique_4_Ezalamalithi | MK427481 |
| psbZ | photosystem II protein Z | Mozambique_5_Calamidade | MK427482 |
| psbZ | photosystem II protein Z | Mozambique_8_Calamidade | MK427483 |
| psbZ | photosystem II protein Z | Mozambique_10_Bwana | MK427484 |
| psbZ | photosystem II protein Z | Mozambique_11_Fernando | MK427485 |
| psbZ | photosystem II protein Z | Mozambique_16_Bwana | MK427486 |
| psbZ | photosystem II protein Z | Mozambique_17_Mulaleia | MK427487 |
| psbZ | photosystem II protein Z | Mozambique_20_Cadri | MK427488 |
| psbZ | photosystem II protein Z | Mozambique_23_Mulaleia | MK427489 |
| psbZ | photosystem II protein Z | Mozambique_Manihot_glaziovii | MK427490 |
| rps14 | ribosomal protein S14 | Tanzania_Healthy | MK427491 |
| rps14 | ribosomal protein S14 | Tanzania_03_Rumara | MK427492 |
| rps14 | ribosomal protein S14 | Tanzania_04_Kibandameno | MK427493 |
| rps14 | ribosomal protein S14 | Tanzania_05_Mkunungu | MK427494 |
| rps14 | ribosomal protein S14 | Tanzania_06_Kibandameno | MK427495 |
| rps14 | ribosomal protein S14 | Tanzania_07_Unknown | MK427496 |
| rps14 | ribosomal protein S14 | Tanzania_08_Unknown | MK427497 |
| rps14 | ribosomal protein S14 | Tanzania_09_Mkangawandu | MK427498 |
| rps14 | ribosomal protein S14 | Tanzania_10_Kibembe | MK427499 |
| rps14 | ribosomal protein S14 | Tanzania_11_Kibembe | MK427500 |
| rps14 | ribosomal protein S14 | Tanzania_12_Mwarabu | MK427501 |
| rps14 | ribosomal protein S14 | Kenya_01_Local | MK427502 |
| rps14 | ribosomal protein S14 | Kenya_02_Megana | MK427503 |
| rps14 | ribosomal protein S14 | Kenya_03_Tereka | MK427504 |
| rps14 | ribosomal protein S14 | Kenya_04_Megana | MK427505 |
| rps14 | ribosomal protein S14 | Kenya_05_Megana | MK427506 |
| rps14 | ribosomal protein S14 | Kenya_07_Local | MK427507 |
| rps14 | ribosomal protein S14 | Kenya_10_Local | MK427508 |
| rps14 | ribosomal protein S14 | Kenya_11_Kibandameno | MK427509 |
| rps14 | ribosomal protein S14 | Kenya_12_Local | MK427510 |
| rps14 | ribosomal protein S14 | Kenya_13_Kibandameno | MK427511 |
| rps14 | ribosomal protein S14 | Kenya_14_Kibandameno | MK427512 |
| rps14 | ribosomal protein S14 | Kenya_15_Kibandameno | MK427513 |
| rps14 | ribosomal protein S14 | Mozambique_4_Ezalamalithi | MK427514 |
| rps14 | ribosomal protein S14 | Mozambique_5_Calamidade | MK427515 |
| rps14 | ribosomal protein S14 | Mozambique_8_Calamidade | MK427516 |
| rps14 | ribosomal protein S14 | Mozambique_10_Bwana | MK427517 |
| rps14 | ribosomal protein S14 | Mozambique_11_Fernando | MK427518 |
| rps14 | ribosomal protein S14 | Mozambique_16_Bwana | MK427519 |
| rps14 | ribosomal protein S14 | Mozambique_17_Mulaleia | MK427520 |
| rps14 | ribosomal protein S14 | Mozambique_20_Cadri | MK427521 |
| rps14 | ribosomal protein S14 | Mozambique_23_Mulaleia | MK427522 |
| rps14 | ribosomal protein S14 | Mozambique_Manihot_glaziovii | MK427523 |
| psaB | photosystem I P700 apoprotein A2 | Tanzania_Healthy | MK427524 |
| psaB | photosystem I P700 apoprotein A2 | Tanzania_03_Rumara | MK427525 |
| psaB | photosystem I P700 apoprotein A2 | Tanzania_04_Kibandameno | MK427526 |
| psaB | photosystem I P700 apoprotein A2 | Tanzania_05_Mkunungu | MK427527 |
| psaB | photosystem I P700 apoprotein A2 | Tanzania_06_Kibandameno | MK427528 |
| psaB | photosystem I P700 apoprotein A2 | Tanzania_07_Unknown | MK427529 |
| psaB | photosystem I P700 apoprotein A2 | Tanzania_08_Unknown | MK427530 |
| psaB | photosystem I P700 apoprotein A2 | Tanzania_09_Mkangawandu | MK427531 |
| psaB | photosystem I P700 apoprotein A2 | Tanzania_10_Kibembe | MK427532 |
| psaB | photosystem I P700 apoprotein A2 | Tanzania_11_Kibembe | MK427533 |
| psaB | photosystem I P700 apoprotein A2 | Tanzania_12_Mwarabu | MK427534 |
| psaB | photosystem I P700 apoprotein A2 | Kenya_01_Local | MK427535 |
| psaB | photosystem I P700 apoprotein A2 | Kenya_02_Megana | MK427536 |
| psaB | photosystem I P700 apoprotein A2 | Kenya_03_Tereka | MK427537 |
| psaB | photosystem I P700 apoprotein A2 | Kenya_04_Megana | MK427538 |
| psaB | photosystem I P700 apoprotein A2 | Kenya_05_Megana | MK427539 |
| psaB | photosystem I P700 apoprotein A2 | Kenya_07_Local | MK427540 |
| psaB | photosystem I P700 apoprotein A2 | Kenya_10_Local | MK427541 |
| psaB | photosystem I P700 apoprotein A2 | Kenya_11_Kibandameno | MK427542 |
| psaB | photosystem I P700 apoprotein A2 | Kenya_12_Local | MK427543 |
| psaB | photosystem I P700 apoprotein A2 | Kenya_13_Kibandameno | MK427544 |
| psaB | photosystem I P700 apoprotein A2 | Kenya_14_Kibandameno | MK427545 |
| psaB | photosystem I P700 apoprotein A2 | Kenya_15_Kibandameno | MK427546 |
| psaB | photosystem I P700 apoprotein A2 | Mozambique_4_Ezalamalithi | MK427547 |
| psaB | photosystem I P700 apoprotein A2 | Mozambique_5_Calamidade | MK427548 |
| psaB | photosystem I P700 apoprotein A2 | Mozambique_8_Calamidade | MK427549 |
| psaB | photosystem I P700 apoprotein A2 | Mozambique_10_Bwana | MK427550 |
| psaB | photosystem I P700 apoprotein A2 | Mozambique_11_Fernando | MK427551 |
| psaB | photosystem I P700 apoprotein A2 | Mozambique_16_Bwana | MK427552 |
| psaB | photosystem I P700 apoprotein A2 | Mozambique_17_Mulaleia | MK427553 |
| psaB | photosystem I P700 apoprotein A2 | Mozambique_20_Cadri | MK427554 |
| psaB | photosystem I P700 apoprotein A2 | Mozambique_23_Mulaleia | MK427555 |
| psaB | photosystem I P700 apoprotein A2 | Mozambique_Manihot_glaziovii | MK427556 |
| psaA | photosystem I P700 apoprotein A1 | Tanzania_Healthy | MK427557 |
| psaA | photosystem I P700 apoprotein A1 | Tanzania_03_Rumara | MK427558 |
| psaA | photosystem I P700 apoprotein A1 | Tanzania_04_Kibandameno | MK427559 |
| psaA | photosystem I P700 apoprotein A1 | Tanzania_05_Mkunungu | MK427560 |
| psaA | photosystem I P700 apoprotein A1 | Tanzania_06_Kibandameno | MK427561 |
| psaA | photosystem I P700 apoprotein A1 | Tanzania_07_Unknown | MK427562 |
| psaA | photosystem I P700 apoprotein A1 | Tanzania_08_Unknown | MK427563 |
| psaA | photosystem I P700 apoprotein A1 | Tanzania_09_Mkangawandu | MK427564 |
| psaA | photosystem I P700 apoprotein A1 | Tanzania_10_Kibembe | MK427565 |
| psaA | photosystem I P700 apoprotein A1 | Tanzania_11_Kibembe | MK427566 |
| psaA | photosystem I P700 apoprotein A1 | Tanzania_12_Mwarabu | MK427567 |
| psaA | photosystem I P700 apoprotein A1 | Kenya_01_Local | MK427568 |
| psaA | photosystem I P700 apoprotein A1 | Kenya_02_Megana | MK427569 |
| psaA | photosystem I P700 apoprotein A1 | Kenya_03_Tereka | MK427570 |
| psaA | photosystem I P700 apoprotein A1 | Kenya_04_Megana | MK427571 |
| psaA | photosystem I P700 apoprotein A1 | Kenya_05_Megana | MK427572 |
| psaA | photosystem I P700 apoprotein A1 | Kenya_07_Local | MK427573 |
| psaA | photosystem I P700 apoprotein A1 | Kenya_10_Local | MK427574 |
| psaA | photosystem I P700 apoprotein A1 | Kenya_11_Kibandameno | MK427575 |
| psaA | photosystem I P700 apoprotein A1 | Kenya_12_Local | MK427576 |
| psaA | photosystem I P700 apoprotein A1 | Kenya_13_Kibandameno | MK427577 |
| psaA | photosystem I P700 apoprotein A1 | Kenya_14_Kibandameno | MK427578 |
| psaA | photosystem I P700 apoprotein A1 | Kenya_15_Kibandameno | MK427579 |
| psaA | photosystem I P700 apoprotein A1 | Mozambique_4_Ezalamalithi | MK427580 |
| psaA | photosystem I P700 apoprotein A1 | Mozambique_5_Calamidade | MK427581 |
| psaA | photosystem I P700 apoprotein A1 | Mozambique_8_Calamidade | MK427582 |
| psaA | photosystem I P700 apoprotein A1 | Mozambique_10_Bwana | MK427583 |
| psaA | photosystem I P700 apoprotein A1 | Mozambique_11_Fernando | MK427584 |
| psaA | photosystem I P700 apoprotein A1 | Mozambique_16_Bwana | MK427585 |
| psaA | photosystem I P700 apoprotein A1 | Mozambique_17_Mulaleia | MK427586 |
| psaA | photosystem I P700 apoprotein A1 | Mozambique_20_Cadri | MK427587 |
| psaA | photosystem I P700 apoprotein A1 | Mozambique_23_Mulaleia | MK427588 |
| psaA | photosystem I P700 apoprotein A1 | Mozambique_Manihot_glaziovii | MK427589 |
| rps4 | ribosomal protein S4 | Tanzania_Healthy | MK427590 |
| rps4 | ribosomal protein S4 | Tanzania_03_Rumara | MK427591 |
| rps4 | ribosomal protein S4 | Tanzania_04_Kibandameno | MK427592 |
| rps4 | ribosomal protein S4 | Tanzania_05_Mkunungu | MK427593 |
| rps4 | ribosomal protein S4 | Tanzania_06_Kibandameno | MK427594 |
| rps4 | ribosomal protein S4 | Tanzania_07_Unknown | MK427595 |
| rps4 | ribosomal protein S4 | Tanzania_08_Unknown | MK427596 |
| rps4 | ribosomal protein S4 | Tanzania_09_Mkangawandu | MK427597 |
| rps4 | ribosomal protein S4 | Tanzania_10_Kibembe | MK427598 |
| rps4 | ribosomal protein S4 | Tanzania_11_Kibembe | MK427599 |
| rps4 | ribosomal protein S4 | Tanzania_12_Mwarabu | MK427600 |
| rps4 | ribosomal protein S4 | Kenya_01_Local | MK427601 |
| rps4 | ribosomal protein S4 | Kenya_02_Megana | MK427602 |
| rps4 | ribosomal protein S4 | Kenya_03_Tereka | MK427603 |
| rps4 | ribosomal protein S4 | Kenya_04_Megana | MK427604 |
| rps4 | ribosomal protein S4 | Kenya_05_Megana | MK427605 |
| rps4 | ribosomal protein S4 | Kenya_07_Local | MK427606 |
| rps4 | ribosomal protein S4 | Kenya_10_Local | MK427607 |
| rps4 | ribosomal protein S4 | Kenya_11_Kibandameno | MK427608 |
| rps4 | ribosomal protein S4 | Kenya_12_Local | MK427609 |
| rps4 | ribosomal protein S4 | Kenya_13_Kibandameno | MK427610 |
| rps4 | ribosomal protein S4 | Kenya_14_Kibandameno | MK427611 |
| rps4 | ribosomal protein S4 | Kenya_15_Kibandameno | MK427612 |
| rps4 | ribosomal protein S4 | Mozambique_4_Ezalamalithi | MK427613 |
| rps4 | ribosomal protein S4 | Mozambique_5_Calamidade | MK427614 |
| rps4 | ribosomal protein S4 | Mozambique_8_Calamidade | MK427615 |
| rps4 | ribosomal protein S4 | Mozambique_10_Bwana | MK427616 |
| rps4 | ribosomal protein S4 | Mozambique_11_Fernando | MK427617 |
| rps4 | ribosomal protein S4 | Mozambique_16_Bwana | MK427618 |
| rps4 | ribosomal protein S4 | Mozambique_17_Mulaleia | MK427619 |
| rps4 | ribosomal protein S4 | Mozambique_20_Cadri | MK427620 |
| rps4 | ribosomal protein S4 | Mozambique_23_Mulaleia | MK427621 |
| rps4 | ribosomal protein S4 | Mozambique_Manihot_glaziovii | MK427622 |
| ndhJ | NADH-plastoquinone oxidoreductase subunit J | Tanzania_Healthy | MK427623 |
| ndhJ | NADH-plastoquinone oxidoreductase subunit J | Tanzania_03_Rumara | MK427624 |
| ndhJ | NADH-plastoquinone oxidoreductase subunit J | Tanzania_04_Kibandameno | MK427625 |
| ndhJ | NADH-plastoquinone oxidoreductase subunit J | Tanzania_05_Mkunungu | MK427626 |
| ndhJ | NADH-plastoquinone oxidoreductase subunit J | Tanzania_06_Kibandameno | MK427627 |
| ndhJ | NADH-plastoquinone oxidoreductase subunit J | Tanzania_07_Unknown | MK427628 |
| ndhJ | NADH-plastoquinone oxidoreductase subunit J | Tanzania_08_Unknown | MK427629 |
| ndhJ | NADH-plastoquinone oxidoreductase subunit J | Tanzania_09_Mkangawandu | MK427630 |
| ndhJ | NADH-plastoquinone oxidoreductase subunit J | Tanzania_10_Kibembe | MK427631 |
| ndhJ | NADH-plastoquinone oxidoreductase subunit J | Tanzania_11_Kibembe | MK427632 |
| ndhJ | NADH-plastoquinone oxidoreductase subunit J | Tanzania_12_Mwarabu | MK427633 |
| ndhJ | NADH-plastoquinone oxidoreductase subunit J | Kenya_01_Local | MK427634 |
| ndhJ | NADH-plastoquinone oxidoreductase subunit J | Kenya_02_Megana | MK427635 |
| ndhJ | NADH-plastoquinone oxidoreductase subunit J | Kenya_03_Tereka | MK427636 |
| ndhJ | NADH-plastoquinone oxidoreductase subunit J | Kenya_04_Megana | MK427637 |
| ndhJ | NADH-plastoquinone oxidoreductase subunit J | Kenya_05_Megana | MK427638 |
| ndhJ | NADH-plastoquinone oxidoreductase subunit J | Kenya_07_Local | MK427639 |
| ndhJ | NADH-plastoquinone oxidoreductase subunit J | Kenya_10_Local | MK427640 |
| ndhJ | NADH-plastoquinone oxidoreductase subunit J | Kenya_11_Kibandameno | MK427641 |
| ndhJ | NADH-plastoquinone oxidoreductase subunit J | Kenya_12_Local | MK427642 |
| ndhJ | NADH-plastoquinone oxidoreductase subunit J | Kenya_13_Kibandameno | MK427643 |
| ndhJ | NADH-plastoquinone oxidoreductase subunit J | Kenya_14_Kibandameno | MK427644 |
| ndhJ | NADH-plastoquinone oxidoreductase subunit J | Kenya_15_Kibandameno | MK427645 |
| ndhJ | NADH-plastoquinone oxidoreductase subunit J | Mozambique_4_Ezalamalithi | MK427646 |
| ndhJ | NADH-plastoquinone oxidoreductase subunit J | Mozambique_5_Calamidade | MK427647 |
| ndhJ | NADH-plastoquinone oxidoreductase subunit J | Mozambique_8_Calamidade | MK427648 |
| ndhJ | NADH-plastoquinone oxidoreductase subunit J | Mozambique_10_Bwana | MK427649 |
| ndhJ | NADH-plastoquinone oxidoreductase subunit J | Mozambique_11_Fernando | MK427650 |
| ndhJ | NADH-plastoquinone oxidoreductase subunit J | Mozambique_16_Bwana | MK427651 |
| ndhJ | NADH-plastoquinone oxidoreductase subunit J | Mozambique_17_Mulaleia | MK427652 |
| ndhJ | NADH-plastoquinone oxidoreductase subunit J | Mozambique_20_Cadri | MK427653 |
| ndhJ | NADH-plastoquinone oxidoreductase subunit J | Mozambique_23_Mulaleia | MK427654 |
| ndhJ | NADH-plastoquinone oxidoreductase subunit J | Mozambique_Manihot_glaziovii | MK427655 |
| ndhK | NADH-plastoquinone oxidoreductase subunit K | Tanzania_Healthy | MK427656 |
| ndhK | NADH-plastoquinone oxidoreductase subunit K | Tanzania_03_Rumara | MK427657 |
| ndhK | NADH-plastoquinone oxidoreductase subunit K | Tanzania_04_Kibandameno | MK427658 |
| ndhK | NADH-plastoquinone oxidoreductase subunit K | Tanzania_05_Mkunungu | MK427659 |
| ndhK | NADH-plastoquinone oxidoreductase subunit K | Tanzania_06_Kibandameno | MK427660 |
| ndhK | NADH-plastoquinone oxidoreductase subunit K | Tanzania_07_Unknown | MK427661 |
| ndhK | NADH-plastoquinone oxidoreductase subunit K | Tanzania_08_Unknown | MK427662 |
| ndhK | NADH-plastoquinone oxidoreductase subunit K | Tanzania_09_Mkangawandu | MK427663 |
| ndhK | NADH-plastoquinone oxidoreductase subunit K | Tanzania_10_Kibembe | MK427664 |
| ndhK | NADH-plastoquinone oxidoreductase subunit K | Tanzania_11_Kibembe | MK427665 |
| ndhK | NADH-plastoquinone oxidoreductase subunit K | Tanzania_12_Mwarabu | MK427666 |
| ndhK | NADH-plastoquinone oxidoreductase subunit K | Kenya_01_Local | MK427667 |
| ndhK | NADH-plastoquinone oxidoreductase subunit K | Kenya_02_Megana | MK427668 |
| ndhK | NADH-plastoquinone oxidoreductase subunit K | Kenya_03_Tereka | MK427669 |
| ndhK | NADH-plastoquinone oxidoreductase subunit K | Kenya_04_Megana | MK427670 |
| ndhK | NADH-plastoquinone oxidoreductase subunit K | Kenya_05_Megana | MK427671 |
| ndhK | NADH-plastoquinone oxidoreductase subunit K | Kenya_07_Local | MK427672 |
| ndhK | NADH-plastoquinone oxidoreductase subunit K | Kenya_10_Local | MK427673 |
| ndhK | NADH-plastoquinone oxidoreductase subunit K | Kenya_11_Kibandameno | MK427674 |
| ndhK | NADH-plastoquinone oxidoreductase subunit K | Kenya_12_Local | MK427675 |
| ndhK | NADH-plastoquinone oxidoreductase subunit K | Kenya_13_Kibandameno | MK427676 |
| ndhK | NADH-plastoquinone oxidoreductase subunit K | Kenya_14_Kibandameno | MK427677 |
| ndhK | NADH-plastoquinone oxidoreductase subunit K | Kenya_15_Kibandameno | MK427678 |
| ndhK | NADH-plastoquinone oxidoreductase subunit K | Mozambique_4_Ezalamalithi | MK427679 |
| ndhK | NADH-plastoquinone oxidoreductase subunit K | Mozambique_5_Calamidade | MK427680 |
| ndhK | NADH-plastoquinone oxidoreductase subunit K | Mozambique_8_Calamidade | MK427681 |
| ndhK | NADH-plastoquinone oxidoreductase subunit K | Mozambique_10_Bwana | MK427682 |
| ndhK | NADH-plastoquinone oxidoreductase subunit K | Mozambique_11_Fernando | MK427683 |
| ndhK | NADH-plastoquinone oxidoreductase subunit K | Mozambique_16_Bwana | MK427684 |
| ndhK | NADH-plastoquinone oxidoreductase subunit K | Mozambique_17_Mulaleia | MK427685 |
| ndhK | NADH-plastoquinone oxidoreductase subunit K | Mozambique_20_Cadri | MK427686 |
| ndhK | NADH-plastoquinone oxidoreductase subunit K | Mozambique_23_Mulaleia | MK427687 |
| ndhK | NADH-plastoquinone oxidoreductase subunit K | Mozambique_Manihot_glaziovii | MK427688 |
| ndhC | NADH-plastoquinone oxidoreductase subunit 3 | Tanzania_Healthy | MK430183 |
| ndhC | NADH-plastoquinone oxidoreductase subunit 3 | Tanzania_04_Kibandameno | MK430185 |
| ndhC | NADH-plastoquinone oxidoreductase subunit 3 | Tanzania_05_Mkunungu | MK430186 |
| ndhC | NADH-plastoquinone oxidoreductase subunit 3 | Tanzania_06_Kibandameno | MK430187 |
| ndhC | NADH-plastoquinone oxidoreductase subunit 3 | Tanzania_07_Unknown | MK430188 |
| ndhC | NADH-plastoquinone oxidoreductase subunit 3 | Tanzania_08_Unknown | MK430189 |
| ndhC | NADH-plastoquinone oxidoreductase subunit 3 | Tanzania_09_Mkangawandu | MK430190 |
| ndhC | NADH-plastoquinone oxidoreductase subunit 3 | Tanzania_10_Kibembe | MK430191 |
| ndhC | NADH-plastoquinone oxidoreductase subunit 3 | Tanzania_11_Kibembe | MK430192 |
| ndhC | NADH-plastoquinone oxidoreductase subunit 3 | Tanzania_12_Mwarabu | MK430193 |
| ndhC | NADH-plastoquinone oxidoreductase subunit 3 | Kenya_01_Local | MK430194 |
| ndhC | NADH-plastoquinone oxidoreductase subunit 3 | Kenya_02_Megana | MK430195 |
| ndhC | NADH-plastoquinone oxidoreductase subunit 3 | Kenya_03_Tereka | MK430196 |
| ndhC | NADH-plastoquinone oxidoreductase subunit 3 | Kenya_04_Megana | MK430197 |
| ndhC | NADH-plastoquinone oxidoreductase subunit 3 | Kenya_05_Megana | MK430198 |
| ndhC | NADH-plastoquinone oxidoreductase subunit 3 | Kenya_07_Local | MK430199 |
| ndhC | NADH-plastoquinone oxidoreductase subunit 3 | Kenya_10_Local | MK430200 |
| ndhC | NADH-plastoquinone oxidoreductase subunit 3 | Kenya_11_Kibandameno | MK430201 |
| ndhC | NADH-plastoquinone oxidoreductase subunit 3 | Kenya_12_Local | MK430202 |
| ndhC | NADH-plastoquinone oxidoreductase subunit 3 | Kenya_13_Kibandameno | MK430203 |
| ndhC | NADH-plastoquinone oxidoreductase subunit 3 | Kenya_14_Kibandameno | MK430204 |
| ndhC | NADH-plastoquinone oxidoreductase subunit 3 | Kenya_15_Kibandameno | MK430205 |
| ndhC | NADH-plastoquinone oxidoreductase subunit 3 | Mozambique_4_Ezalamalithi | MK430206 |
| ndhC | NADH-plastoquinone oxidoreductase subunit 3 | Mozambique_5_Calamidade | MK430207 |
| ndhC | NADH-plastoquinone oxidoreductase subunit 3 | Mozambique_8_Calamidade | MK430208 |
| ndhC | NADH-plastoquinone oxidoreductase subunit 3 | Mozambique_10_Bwana | MK430209 |
| ndhC | NADH-plastoquinone oxidoreductase subunit 3 | Mozambique_11_Fernando | MK430210 |
| ndhC | NADH-plastoquinone oxidoreductase subunit 3 | Mozambique_16_Bwana | MK430211 |
| ndhC | NADH-plastoquinone oxidoreductase subunit 3 | Mozambique_17_Mulaleia | MK430212 |
| ndhC | NADH-plastoquinone oxidoreductase subunit 3 | Mozambique_20_Cadri | MK430213 |
| ndhC | NADH-plastoquinone oxidoreductase subunit 3 | Mozambique_23_Mulaleia | MK430214 |
| ndhC | NADH-plastoquinone oxidoreductase subunit 3 | Mozambique_Manihot_glaziovii | MK430215 |
| atpE | ATP synthase CF1 epsilon subunit | Tanzania_Healthy | MK430216 |
| atpE | ATP synthase CF1 epsilon subunit | Tanzania_03_Rumara | MK430217 |
| atpE | ATP synthase CF1 epsilon subunit | Tanzania_04_Kibandameno | MK430218 |
| atpE | ATP synthase CF1 epsilon subunit | Tanzania_05_Mkunungu | MK430219 |
| atpE | ATP synthase CF1 epsilon subunit | Tanzania_06_Kibandameno | MK430220 |
| atpE | ATP synthase CF1 epsilon subunit | Tanzania_07_Unknown | MK430221 |
| atpE | ATP synthase CF1 epsilon subunit | Tanzania_08_Unknown | MK430222 |
| atpE | ATP synthase CF1 epsilon subunit | Tanzania_09_Mkangawandu | MK430223 |
| atpE | ATP synthase CF1 epsilon subunit | Tanzania_10_Kibembe | MK430224 |
| atpE | ATP synthase CF1 epsilon subunit | Tanzania_11_Kibembe | MK430225 |
| atpE | ATP synthase CF1 epsilon subunit | Tanzania_12_Mwarabu | MK430226 |
| atpE | ATP synthase CF1 epsilon subunit | Kenya_01_Local | MK430227 |
| atpE | ATP synthase CF1 epsilon subunit | Kenya_02_Megana | MK430228 |
| atpE | ATP synthase CF1 epsilon subunit | Kenya_03_Tereka | MK430229 |
| atpE | ATP synthase CF1 epsilon subunit | Kenya_04_Megana | MK430230 |
| atpE | ATP synthase CF1 epsilon subunit | Kenya_05_Megana | MK430231 |
| atpE | ATP synthase CF1 epsilon subunit | Kenya_07_Local | MK430232 |
| atpE | ATP synthase CF1 epsilon subunit | Kenya_10_Local | MK430233 |
| atpE | ATP synthase CF1 epsilon subunit | Kenya_11_Kibandameno | MK430234 |
| atpE | ATP synthase CF1 epsilon subunit | Kenya_12_Local | MK430235 |
| atpE | ATP synthase CF1 epsilon subunit | Kenya_13_Kibandameno | MK430236 |
| atpE | ATP synthase CF1 epsilon subunit | Kenya_14_Kibandameno | MK430237 |
| atpE | ATP synthase CF1 epsilon subunit | Kenya_15_Kibandameno | MK430238 |
| atpE | ATP synthase CF1 epsilon subunit | Mozambique_4_Ezalamalithi | MK430239 |
| atpE | ATP synthase CF1 epsilon subunit | Mozambique_5_Calamidade | MK430240 |
| atpE | ATP synthase CF1 epsilon subunit | Mozambique_8_Calamidade | MK430241 |
| atpE | ATP synthase CF1 epsilon subunit | Mozambique_10_Bwana | MK430242 |
| atpE | ATP synthase CF1 epsilon subunit | Mozambique_11_Fernando | MK430243 |
| atpE | ATP synthase CF1 epsilon subunit | Mozambique_16_Bwana | MK430244 |
| atpE | ATP synthase CF1 epsilon subunit | Mozambique_17_Mulaleia | MK430245 |
| atpE | ATP synthase CF1 epsilon subunit | Mozambique_20_Cadri | MK430246 |
| atpE | ATP synthase CF1 epsilon subunit | Mozambique_23_Mulaleia | MK430247 |
| atpE | ATP synthase CF1 epsilon subunit | Mozambique_Manihot_glaziovii | MK430248 |
| atpB | ATP synthase CF1 beta subunit | Tanzania_Healthy | MK430249 |
| atpB | ATP synthase CF1 beta subunit | Tanzania_03_Rumara | MK430250 |
| atpB | ATP synthase CF1 beta subunit | Tanzania_04_Kibandameno | MK430251 |
| atpB | ATP synthase CF1 beta subunit | Tanzania_05_Mkunungu | MK430252 |
| atpB | ATP synthase CF1 beta subunit | Tanzania_06_Kibandameno | MK430253 |
| atpB | ATP synthase CF1 beta subunit | Tanzania_07_Unknown | MK430254 |
| atpB | ATP synthase CF1 beta subunit | Tanzania_08_Unknown | MK430255 |
| atpB | ATP synthase CF1 beta subunit | Tanzania_09_Mkangawandu | MK430256 |
| atpB | ATP synthase CF1 beta subunit | Tanzania_10_Kibembe | MK430257 |
| atpB | ATP synthase CF1 beta subunit | Tanzania_11_Kibembe | MK430258 |
| atpB | ATP synthase CF1 beta subunit | Tanzania_12_Mwarabu | MK430259 |
| atpB | ATP synthase CF1 beta subunit | Kenya_01_Local | MK430260 |
| atpB | ATP synthase CF1 beta subunit | Kenya_02_Megana | MK430261 |
| atpB | ATP synthase CF1 beta subunit | Kenya_03_Tereka | MK430262 |
| atpB | ATP synthase CF1 beta subunit | Kenya_04_Megana | MK430263 |
| atpB | ATP synthase CF1 beta subunit | Kenya_05_Megana | MK430264 |
| atpB | ATP synthase CF1 beta subunit | Kenya_07_Local | MK430265 |
| atpB | ATP synthase CF1 beta subunit | Kenya_10_Local | MK430266 |
| atpB | ATP synthase CF1 beta subunit | Kenya_11_Kibandameno | MK430267 |
| atpB | ATP synthase CF1 beta subunit | Kenya_12_Local | MK430268 |
| atpB | ATP synthase CF1 beta subunit | Kenya_13_Kibandameno | MK430269 |
| atpB | ATP synthase CF1 beta subunit | Kenya_14_Kibandameno | MK430270 |
| atpB | ATP synthase CF1 beta subunit | Kenya_15_Kibandameno | MK430271 |
| atpB | ATP synthase CF1 beta subunit | Mozambique_4_Ezalamalithi | MK430272 |
| atpB | ATP synthase CF1 beta subunit | Mozambique_5_Calamidade | MK430273 |
| atpB | ATP synthase CF1 beta subunit | Mozambique_8_Calamidade | MK430274 |
| atpB | ATP synthase CF1 beta subunit | Mozambique_10_Bwana | MK430275 |
| atpB | ATP synthase CF1 beta subunit | Mozambique_11_Fernando | MK430276 |
| atpB | ATP synthase CF1 beta subunit | Mozambique_16_Bwana | MK430277 |
| atpB | ATP synthase CF1 beta subunit | Mozambique_17_Mulaleia | MK430278 |
| atpB | ATP synthase CF1 beta subunit | Mozambique_20_Cadri | MK430279 |
| atpB | ATP synthase CF1 beta subunit | Mozambique_23_Mulaleia | MK430280 |
| atpB | ATP synthase CF1 beta subunit | Mozambique_Manihot_glaziovii | MK430281 |
| rbcL | ribulose-1,5-bisphosphate carboxylase/oxygenase large subunit | Tanzania_Healthy | MK430282 |
| rbcL | ribulose-1,5-bisphosphate carboxylase/oxygenase large subunit | Tanzania_03_Rumara | MK430283 |
| rbcL | ribulose-1,5-bisphosphate carboxylase/oxygenase large subunit | Tanzania_04_Kibandameno | MK430284 |
| rbcL | ribulose-1,5-bisphosphate carboxylase/oxygenase large subunit | Tanzania_05_Mkunungu | MK430285 |
| rbcL | ribulose-1,5-bisphosphate carboxylase/oxygenase large subunit | Tanzania_06_Kibandameno | MK430286 |
| rbcL | ribulose-1,5-bisphosphate carboxylase/oxygenase large subunit | Tanzania_07_Unknown | MK430287 |
| rbcL | ribulose-1,5-bisphosphate carboxylase/oxygenase large subunit | Tanzania_08_Unknown | MK430288 |
| rbcL | ribulose-1,5-bisphosphate carboxylase/oxygenase large subunit | Tanzania_09_Mkangawandu | MK430289 |
| rbcL | ribulose-1,5-bisphosphate carboxylase/oxygenase large subunit | Tanzania_10_Kibembe | MK430290 |
| rbcL | ribulose-1,5-bisphosphate carboxylase/oxygenase large subunit | Tanzania_11_Kibembe | MK430291 |
| rbcL | ribulose-1,5-bisphosphate carboxylase/oxygenase large subunit | Tanzania_12_Mwarabu | MK430292 |
| rbcL | ribulose-1,5-bisphosphate carboxylase/oxygenase large subunit | Kenya_01_Local | MK430293 |
| rbcL | ribulose-1,5-bisphosphate carboxylase/oxygenase large subunit | Kenya_02_Megana | MK430294 |
| rbcL | ribulose-1,5-bisphosphate carboxylase/oxygenase large subunit | Kenya_03_Tereka | MK430295 |
| rbcL | ribulose-1,5-bisphosphate carboxylase/oxygenase large subunit | Kenya_04_Megana | MK430296 |
| rbcL | ribulose-1,5-bisphosphate carboxylase/oxygenase large subunit | Kenya_05_Megana | MK430297 |
| rbcL | ribulose-1,5-bisphosphate carboxylase/oxygenase large subunit | Kenya_07_Local | MK430298 |
| rbcL | ribulose-1,5-bisphosphate carboxylase/oxygenase large subunit | Kenya_10_Local | MK430299 |
| rbcL | ribulose-1,5-bisphosphate carboxylase/oxygenase large subunit | Kenya_11_Kibandameno | MK430300 |
| rbcL | ribulose-1,5-bisphosphate carboxylase/oxygenase large subunit | Kenya_12_Local | MK430301 |
| rbcL | ribulose-1,5-bisphosphate carboxylase/oxygenase large subunit | Kenya_13_Kibandameno | MK430302 |
| rbcL | ribulose-1,5-bisphosphate carboxylase/oxygenase large subunit | Kenya_14_Kibandameno | MK430303 |
| rbcL | ribulose-1,5-bisphosphate carboxylase/oxygenase large subunit | Kenya_15_Kibandameno | MK430304 |
| rbcL | ribulose-1,5-bisphosphate carboxylase/oxygenase large subunit | Mozambique_4_Ezalamalithi | MK430305 |
| rbcL | ribulose-1,5-bisphosphate carboxylase/oxygenase large subunit | Mozambique_5_Calamidade | MK430306 |
| rbcL | ribulose-1,5-bisphosphate carboxylase/oxygenase large subunit | Mozambique_8_Calamidade | MK430307 |
| rbcL | ribulose-1,5-bisphosphate carboxylase/oxygenase large subunit | Mozambique_10_Bwana | MK430308 |
| rbcL | ribulose-1,5-bisphosphate carboxylase/oxygenase large subunit | Mozambique_11_Fernando | MK430309 |
| rbcL | ribulose-1,5-bisphosphate carboxylase/oxygenase large subunit | Mozambique_16_Bwana | MK430310 |
| rbcL | ribulose-1,5-bisphosphate carboxylase/oxygenase large subunit | Mozambique_17_Mulaleia | MK430311 |
| rbcL | ribulose-1,5-bisphosphate carboxylase/oxygenase large subunit | Mozambique_20_Cadri | MK430312 |
| rbcL | ribulose-1,5-bisphosphate carboxylase/oxygenase large subunit | Mozambique_23_Mulaleia | MK430313 |
| rbcL | ribulose-1,5-bisphosphate carboxylase/oxygenase large subunit | Mozambique_Manihot_glaziovii | MK430314 |
| psaI | photosystem I subunit VIII | Tanzania_Healthy | MK430315 |
| psaI | photosystem I subunit VIII | Tanzania_03_Rumara | MK430316 |
| psaI | photosystem I subunit VIII | Tanzania_04_Kibandameno | MK430317 |
| psaI | photosystem I subunit VIII | Tanzania_05_Mkunungu | MK430318 |
| psaI | photosystem I subunit VIII | Tanzania_06_Kibandameno | MK430319 |
| psaI | photosystem I subunit VIII | Tanzania_07_Unknown | MK430320 |
| psaI | photosystem I subunit VIII | Tanzania_08_Unknown | MK430321 |
| psaI | photosystem I subunit VIII | Tanzania_09_Mkangawandu | MK430322 |
| psaI | photosystem I subunit VIII | Tanzania_10_Kibembe | MK430323 |
| psaI | photosystem I subunit VIII | Tanzania_11_Kibembe | MK430324 |
| psaI | photosystem I subunit VIII | Tanzania_12_Mwarabu | MK430325 |
| psaI | photosystem I subunit VIII | Kenya_01_Local | MK430326 |
| psaI | photosystem I subunit VIII | Kenya_02_Megana | MK430327 |
| psaI | photosystem I subunit VIII | Kenya_03_Tereka | MK430328 |
| psaI | photosystem I subunit VIII | Kenya_04_Megana | MK430329 |
| psaI | photosystem I subunit VIII | Kenya_05_Megana | MK430330 |
| psaI | photosystem I subunit VIII | Kenya_07_Local | MK430331 |
| psaI | photosystem I subunit VIII | Kenya_10_Local | MK430332 |
| psaI | photosystem I subunit VIII | Kenya_11_Kibandameno | MK430333 |
| psaI | photosystem I subunit VIII | Kenya_12_Local | MK430334 |
| psaI | photosystem I subunit VIII | Kenya_13_Kibandameno | MK430335 |
| psaI | photosystem I subunit VIII | Kenya_14_Kibandameno | MK430336 |
| psaI | photosystem I subunit VIII | Kenya_15_Kibandameno | MK430337 |
| psaI | photosystem I subunit VIII | Mozambique_4_Ezalamalithi | MK430338 |
| psaI | photosystem I subunit VIII | Mozambique_5_Calamidade | MK430339 |
| psaI | photosystem I subunit VIII | Mozambique_8_Calamidade | MK430340 |
| psaI | photosystem I subunit VIII | Mozambique_10_Bwana | MK430341 |
| psaI | photosystem I subunit VIII | Mozambique_11_Fernando | MK430342 |
| psaI | photosystem I subunit VIII | Mozambique_16_Bwana | MK430343 |
| psaI | photosystem I subunit VIII | Mozambique_17_Mulaleia | MK430344 |
| psaI | photosystem I subunit VIII | Mozambique_20_Cadri | MK430345 |
| psaI | photosystem I subunit VIII | Mozambique_23_Mulaleia | MK430346 |
| psaI | photosystem I subunit VIII | Mozambique_Manihot_glaziovii | MK430347 |
| cemA | chloroplast envelope membrane protein | Tanzania_Healthy | MK430348 |
| cemA | chloroplast envelope membrane protein | Tanzania_03_Rumara | MK430349 |
| cemA | chloroplast envelope membrane protein | Tanzania_04_Kibandameno | MK430350 |
| cemA | chloroplast envelope membrane protein | Tanzania_05_Mkunungu | MK430351 |
| cemA | chloroplast envelope membrane protein | Tanzania_06_Kibandameno | MK430352 |
| cemA | chloroplast envelope membrane protein | Tanzania_07_Unknown | MK430353 |
| cemA | chloroplast envelope membrane protein | Tanzania_08_Unknown | MK430354 |
| cemA | chloroplast envelope membrane protein | Tanzania_09_Mkangawandu | MK430355 |
| cemA | chloroplast envelope membrane protein | Tanzania_10_Kibembe | MK430356 |
| cemA | chloroplast envelope membrane protein | Tanzania_11_Kibembe | MK430357 |
| cemA | chloroplast envelope membrane protein | Tanzania_12_Mwarabu | MK430358 |
| cemA | chloroplast envelope membrane protein | Kenya_01_Local | MK430359 |
| cemA | chloroplast envelope membrane protein | Kenya_02_Megana | MK430360 |
| cemA | chloroplast envelope membrane protein | Kenya_03_Tereka | MK430361 |
| cemA | chloroplast envelope membrane protein | Kenya_04_Megana | MK430362 |
| cemA | chloroplast envelope membrane protein | Kenya_05_Megana | MK430363 |
| cemA | chloroplast envelope membrane protein | Kenya_07_Local | MK430364 |
| cemA | chloroplast envelope membrane protein | Kenya_10_Local | MK430365 |
| cemA | chloroplast envelope membrane protein | Kenya_11_Kibandameno | MK430366 |
| cemA | chloroplast envelope membrane protein | Kenya_12_Local | MK430367 |
| cemA | chloroplast envelope membrane protein | Kenya_13_Kibandameno | MK430368 |
| cemA | chloroplast envelope membrane protein | Kenya_14_Kibandameno | MK430369 |
| cemA | chloroplast envelope membrane protein | Kenya_15_Kibandameno | MK430370 |
| cemA | chloroplast envelope membrane protein | Mozambique_4_Ezalamalithi | MK430371 |
| cemA | chloroplast envelope membrane protein | Mozambique_5_Calamidade | MK430372 |
| cemA | chloroplast envelope membrane protein | Mozambique_8_Calamidade | MK430373 |
| cemA | chloroplast envelope membrane protein | Mozambique_10_Bwana | MK430374 |
| cemA | chloroplast envelope membrane protein | Mozambique_11_Fernando | MK430375 |
| cemA | chloroplast envelope membrane protein | Mozambique_16_Bwana | MK430376 |
| cemA | chloroplast envelope membrane protein | Mozambique_17_Mulaleia | MK430377 |
| cemA | chloroplast envelope membrane protein | Mozambique_20_Cadri | MK430378 |
| cemA | chloroplast envelope membrane protein | Mozambique_23_Mulaleia | MK430379 |
| cemA | chloroplast envelope membrane protein | Mozambique_Manihot_glaziovii | MK430380 |
| petA | cytochrome f | Tanzania_Healthy | MK430381 |
| petA | cytochrome f | Tanzania_03_Rumara | MK430382 |
| petA | cytochrome f | Tanzania_04_Kibandameno | MK430383 |
| petA | cytochrome f | Tanzania_05_Mkunungu | MK430384 |
| petA | cytochrome f | Tanzania_06_Kibandameno | MK430385 |
| petA | cytochrome f | Tanzania_07_Unknown | MK430386 |
| petA | cytochrome f | Tanzania_08_Unknown | MK430387 |
| petA | cytochrome f | Tanzania_09_Mkangawandu | MK430388 |
| petA | cytochrome f | Tanzania_10_Kibembe | MK430389 |
| petA | cytochrome f | Tanzania_11_Kibembe | MK430390 |
| petA | cytochrome f | Tanzania_12_Mwarabu | MK430391 |
| petA | cytochrome f | Kenya_01_Local | MK430392 |
| petA | cytochrome f | Kenya_02_Megana | MK430393 |
| petA | cytochrome f | Kenya_03_Tereka | MK430394 |
| petA | cytochrome f | Kenya_04_Megana | MK430395 |
| petA | cytochrome f | Kenya_05_Megana | MK430396 |
| petA | cytochrome f | Kenya_07_Local | MK430397 |
| petA | cytochrome f | Kenya_10_Local | MK430398 |
| petA | cytochrome f | Kenya_11_Kibandameno | MK430399 |
| petA | cytochrome f | Kenya_12_Local | MK430400 |
| petA | cytochrome f | Kenya_13_Kibandameno | MK430401 |
| petA | cytochrome f | Kenya_14_Kibandameno | MK430402 |
| petA | cytochrome f | Kenya_15_Kibandameno | MK430403 |
| petA | cytochrome f | Mozambique_4_Ezalamalithi | MK430404 |
| petA | cytochrome f | Mozambique_5_Calamidade | MK430405 |
| petA | cytochrome f | Mozambique_8_Calamidade | MK430406 |
| petA | cytochrome f | Mozambique_10_Bwana | MK430407 |
| petA | cytochrome f | Mozambique_11_Fernando | MK430408 |
| petA | cytochrome f | Mozambique_16_Bwana | MK430409 |
| petA | cytochrome f | Mozambique_17_Mulaleia | MK430410 |
| petA | cytochrome f | Mozambique_20_Cadri | MK430411 |
| petA | cytochrome f | Mozambique_23_Mulaleia | MK430412 |
| petA | cytochrome f | Mozambique_Manihot_glaziovii | MK430413 |
| psbJ | photosystem II protein J | Tanzania_Healthy | MK455191 |
| psbJ | photosystem II protein J | Tanzania_03_Rumara | MK455192 |
| psbJ | photosystem II protein J | Tanzania_04_Kibandameno | MK455193 |
| psbJ | photosystem II protein J | Tanzania_05_Mkunungu | MK455194 |
| psbJ | photosystem II protein J | Tanzania_06_Kibandameno | MK455195 |
| psbJ | photosystem II protein J | Tanzania_07_Unknown | MK455196 |
| psbJ | photosystem II protein J | Tanzania_08_Unknown | MK455197 |
| psbJ | photosystem II protein J | Tanzania_09_Mkangawandu | MK455198 |
| psbJ | photosystem II protein J | Tanzania_10_Kibembe | MK455199 |
| psbJ | photosystem II protein J | Tanzania_11_Kibembe | MK455200 |
| psbJ | photosystem II protein J | Tanzania_12_Mwarabu | MK455201 |
| psbJ | photosystem II protein J | Kenya_01_Local | MK455202 |
| psbJ | photosystem II protein J | Kenya_02_Megana | MK455203 |
| psbJ | photosystem II protein J | Kenya_03_Tereka | MK455204 |
| psbJ | photosystem II protein J | Kenya_04_Megana | MK455205 |
| psbJ | photosystem II protein J | Kenya_05_Megana | MK455206 |
| psbJ | photosystem II protein J | Kenya_07_Local | MK455207 |
| psbJ | photosystem II protein J | Kenya_10_Local | MK455208 |
| psbJ | photosystem II protein J | Kenya_11_Kibandameno | MK455209 |
| psbJ | photosystem II protein J | Kenya_12_Local | MK455210 |
| psbJ | photosystem II protein J | Kenya_13_Kibandameno | MK455211 |
| psbJ | photosystem II protein J | Kenya_14_Kibandameno | MK455212 |
| psbJ | photosystem II protein J | Kenya_15_Kibandameno | MK455213 |
| psbJ | photosystem II protein J | Mozambique_4_Ezalamalithi | MK455214 |
| psbJ | photosystem II protein J | Mozambique_5_Calamidade | MK455215 |
| psbJ | photosystem II protein J | Mozambique_8_Calamidade | MK455216 |
| psbJ | photosystem II protein J | Mozambique_10_Bwana | MK455217 |
| psbJ | photosystem II protein J | Mozambique_11_Fernando | MK455218 |
| psbJ | photosystem II protein J | Mozambique_16_Bwana | MK455219 |
| psbJ | photosystem II protein J | Mozambique_17_Mulaleia | MK455220 |
| psbJ | photosystem II protein J | Mozambique_20_Cadri | MK455221 |
| psbJ | photosystem II protein J | Mozambique_23_Mulaleia | MK455222 |
| psbJ | photosystem II protein J | Mozambique_Manihot_glaziovii | MK455223 |
| psbL | photosystem II protein L | Tanzania_Healthy | MK455224 |
| psbL | photosystem II protein L | Tanzania_03_Rumara | MK455225 |
| psbL | photosystem II protein L | Tanzania_04_Kibandameno | MK455226 |
| psbL | photosystem II protein L | Tanzania_05_Mkunungu | MK455227 |
| psbL | photosystem II protein L | Tanzania_06_Kibandameno | MK455228 |
| psbL | photosystem II protein L | Tanzania_07_Unknown | MK455229 |
| psbL | photosystem II protein L | Tanzania_08_Unknown | MK455230 |
| psbL | photosystem II protein L | Tanzania_09_Mkangawandu | MK455231 |
| psbL | photosystem II protein L | Tanzania_10_Kibembe | MK455232 |
| psbL | photosystem II protein L | Tanzania_11_Kibembe | MK455233 |
| psbL | photosystem II protein L | Tanzania_12_Mwarabu | MK455234 |
| psbL | photosystem II protein L | Kenya_01_Local | MK455235 |
| psbL | photosystem II protein L | Kenya_02_Megana | MK455236 |
| psbL | photosystem II protein L | Kenya_03_Tereka | MK455237 |
| psbL | photosystem II protein L | Kenya_04_Megana | MK455238 |
| psbL | photosystem II protein L | Kenya_05_Megana | MK455239 |
| psbL | photosystem II protein L | Kenya_07_Local | MK455240 |
| psbL | photosystem II protein L | Kenya_10_Local | MK455241 |
| psbL | photosystem II protein L | Kenya_11_Kibandameno | MK455242 |
| psbL | photosystem II protein L | Kenya_12_Local | MK455243 |
| psbL | photosystem II protein L | Kenya_13_Kibandameno | MK455244 |
| psbL | photosystem II protein L | Kenya_14_Kibandameno | MK455245 |
| psbL | photosystem II protein L | Kenya_15_Kibandameno | MK455246 |
| psbL | photosystem II protein L | Mozambique_4_Ezalamalithi | MK455247 |
| psbL | photosystem II protein L | Mozambique_5_Calamidade | MK455248 |
| psbL | photosystem II protein L | Mozambique_8_Calamidade | MK455249 |
| psbL | photosystem II protein L | Mozambique_10_Bwana | MK455250 |
| psbL | photosystem II protein L | Mozambique_11_Fernando | MK455251 |
| psbL | photosystem II protein L | Mozambique_16_Bwana | MK455252 |
| psbL | photosystem II protein L | Mozambique_17_Mulaleia | MK455253 |
| psbL | photosystem II protein L | Mozambique_20_Cadri | MK455254 |
| psbL | photosystem II protein L | Mozambique_23_Mulaleia | MK455255 |
| psbL | photosystem II protein L | Mozambique_Manihot_glaziovii | MK455256 |
| psbF | photosystem II cytochrome b559 beta subunit | Tanzania_Healthy | MK455257 |
| psbF | photosystem II cytochrome b559 beta subunit | Tanzania_03_Rumara | MK455258 |
| psbF | photosystem II cytochrome b559 beta subunit | Tanzania_04_Kibandameno | MK455259 |
| psbF | photosystem II cytochrome b559 beta subunit | Tanzania_05_Mkunungu | MK455260 |
| psbF | photosystem II cytochrome b559 beta subunit | Tanzania_06_Kibandameno | MK455261 |
| psbF | photosystem II cytochrome b559 beta subunit | Tanzania_07_Unknown | MK455262 |
| psbF | photosystem II cytochrome b559 beta subunit | Tanzania_08_Unknown | MK455263 |
| psbF | photosystem II cytochrome b559 beta subunit | Tanzania_09_Mkangawandu | MK455264 |
| psbF | photosystem II cytochrome b559 beta subunit | Tanzania_10_Kibembe | MK455265 |
| psbF | photosystem II cytochrome b559 beta subunit | Tanzania_11_Kibembe | MK455266 |
| psbF | photosystem II cytochrome b559 beta subunit | Tanzania_12_Mwarabu | MK455267 |
| psbF | photosystem II cytochrome b559 beta subunit | Kenya_01_Local | MK455268 |
| psbF | photosystem II cytochrome b559 beta subunit | Kenya_02_Megana | MK455269 |
| psbF | photosystem II cytochrome b559 beta subunit | Kenya_03_Tereka | MK455270 |
| psbF | photosystem II cytochrome b559 beta subunit | Kenya_04_Megana | MK455271 |
| psbF | photosystem II cytochrome b559 beta subunit | Kenya_05_Megana | MK455272 |
| psbF | photosystem II cytochrome b559 beta subunit | Kenya_07_Local | MK455273 |
| psbF | photosystem II cytochrome b559 beta subunit | Kenya_10_Local | MK455274 |
| psbF | photosystem II cytochrome b559 beta subunit | Kenya_11_Kibandameno | MK455275 |
| psbF | photosystem II cytochrome b559 beta subunit | Kenya_12_Local | MK455276 |
| psbF | photosystem II cytochrome b559 beta subunit | Kenya_13_Kibandameno | MK455277 |
| psbF | photosystem II cytochrome b559 beta subunit | Kenya_14_Kibandameno | MK455278 |
| psbF | photosystem II cytochrome b559 beta subunit | Kenya_15_Kibandameno | MK455279 |
| psbF | photosystem II cytochrome b559 beta subunit | Mozambique_4_Ezalamalithi | MK455280 |
| psbF | photosystem II cytochrome b559 beta subunit | Mozambique_5_Calamidade | MK455281 |
| psbF | photosystem II cytochrome b559 beta subunit | Mozambique_8_Calamidade | MK455282 |
| psbF | photosystem II cytochrome b559 beta subunit | Mozambique_10_Bwana | MK455283 |
| psbF | photosystem II cytochrome b559 beta subunit | Mozambique_11_Fernando | MK455284 |
| psbF | photosystem II cytochrome b559 beta subunit | Mozambique_16_Bwana | MK455285 |
| psbF | photosystem II cytochrome b559 beta subunit | Mozambique_17_Mulaleia | MK455286 |
| psbF | photosystem II cytochrome b559 beta subunit | Mozambique_20_Cadri | MK455287 |
| psbF | photosystem II cytochrome b559 beta subunit | Mozambique_23_Mulaleia | MK455288 |
| psbF | photosystem II cytochrome b559 beta subunit | Mozambique_Manihot_glaziovii | MK455289 |
| psbE | photosystem II cytochrome b559 alpha subunit | Tanzania_Healthy | MK455290 |
| psbE | photosystem II cytochrome b559 alpha subunit | Tanzania_03_Rumara | MK455291 |
| psbE | photosystem II cytochrome b559 alpha subunit | Tanzania_04_Kibandameno | MK455292 |
| psbE | photosystem II cytochrome b559 alpha subunit | Tanzania_05_Mkunungu | MK455293 |
| psbE | photosystem II cytochrome b559 alpha subunit | Tanzania_06_Kibandameno | MK455294 |
| psbE | photosystem II cytochrome b559 alpha subunit | Tanzania_07_Unknown | MK455295 |
| psbE | photosystem II cytochrome b559 alpha subunit | Tanzania_08_Unknown | MK455296 |
| psbE | photosystem II cytochrome b559 alpha subunit | Tanzania_09_Mkangawandu | MK455297 |
| psbE | photosystem II cytochrome b559 alpha subunit | Tanzania_10_Kibembe | MK455298 |
| psbE | photosystem II cytochrome b559 alpha subunit | Tanzania_11_Kibembe | MK455299 |
| psbE | photosystem II cytochrome b559 alpha subunit | Tanzania_12_Mwarabu | MK455300 |
| psbE | photosystem II cytochrome b559 alpha subunit | Kenya_01_Local | MK455301 |
| psbE | photosystem II cytochrome b559 alpha subunit | Kenya_02_Megana | MK455302 |
| psbE | photosystem II cytochrome b559 alpha subunit | Kenya_03_Tereka | MK455303 |
| psbE | photosystem II cytochrome b559 alpha subunit | Kenya_04_Megana | MK455304 |
| psbE | photosystem II cytochrome b559 alpha subunit | Kenya_05_Megana | MK455305 |
| psbE | photosystem II cytochrome b559 alpha subunit | Kenya_07_Local | MK455306 |
| psbE | photosystem II cytochrome b559 alpha subunit | Kenya_10_Local | MK455307 |
| psbE | photosystem II cytochrome b559 alpha subunit | Kenya_11_Kibandameno | MK455308 |
| psbE | photosystem II cytochrome b559 alpha subunit | Kenya_12_Local | MK455309 |
| psbE | photosystem II cytochrome b559 alpha subunit | Kenya_13_Kibandameno | MK455310 |
| psbE | photosystem II cytochrome b559 alpha subunit | Kenya_14_Kibandameno | MK455311 |
| psbE | photosystem II cytochrome b559 alpha subunit | Kenya_15_Kibandameno | MK455312 |
| psbE | photosystem II cytochrome b559 alpha subunit | Mozambique_4_Ezalamalithi | MK455313 |
| psbE | photosystem II cytochrome b559 alpha subunit | Mozambique_5_Calamidade | MK455314 |
| psbE | photosystem II cytochrome b559 alpha subunit | Mozambique_8_Calamidade | MK455315 |
| psbE | photosystem II cytochrome b559 alpha subunit | Mozambique_10_Bwana | MK455316 |
| psbE | photosystem II cytochrome b559 alpha subunit | Mozambique_11_Fernando | MK455317 |
| psbE | photosystem II cytochrome b559 alpha subunit | Mozambique_16_Bwana | MK455318 |
| psbE | photosystem II cytochrome b559 alpha subunit | Mozambique_17_Mulaleia | MK455319 |
| psbE | photosystem II cytochrome b559 alpha subunit | Mozambique_20_Cadri | MK455320 |
| psbE | photosystem II cytochrome b559 alpha subunit | Mozambique_23_Mulaleia | MK455321 |
| psbE | photosystem II cytochrome b559 alpha subunit | Mozambique_Manihot_glaziovii | MK455322 |
| petG | cytochrome b6/f complex subunit V | Tanzania_Healthy | MK455323 |
| petG | cytochrome b6/f complex subunit V | Tanzania_03_Rumara | MK455324 |
| petG | cytochrome b6/f complex subunit V | Tanzania_04_Kibandameno | MK455325 |
| petG | cytochrome b6/f complex subunit V | Tanzania_05_Mkunungu | MK455326 |
| petG | cytochrome b6/f complex subunit V | Tanzania_06_Kibandameno | MK455327 |
| petG | cytochrome b6/f complex subunit V | Tanzania_07_Unknown | MK455328 |
| petG | cytochrome b6/f complex subunit V | Tanzania_08_Unknown | MK455329 |
| petG | cytochrome b6/f complex subunit V | Tanzania_09_Mkangawandu | MK455330 |
| petG | cytochrome b6/f complex subunit V | Tanzania_10_Kibembe | MK455331 |
| petG | cytochrome b6/f complex subunit V | Tanzania_11_Kibembe | MK455332 |
| petG | cytochrome b6/f complex subunit V | Tanzania_12_Mwarabu | MK455333 |
| petG | cytochrome b6/f complex subunit V | Kenya_01_Local | MK455334 |
| petG | cytochrome b6/f complex subunit V | Kenya_02_Megana | MK455335 |
| petG | cytochrome b6/f complex subunit V | Kenya_03_Tereka | MK455336 |
| petG | cytochrome b6/f complex subunit V | Kenya_04_Megana | MK455337 |
| petG | cytochrome b6/f complex subunit V | Kenya_05_Megana | MK455338 |
| petG | cytochrome b6/f complex subunit V | Kenya_07_Local | MK455339 |
| petG | cytochrome b6/f complex subunit V | Kenya_10_Local | MK455340 |
| petG | cytochrome b6/f complex subunit V | Kenya_11_Kibandameno | MK455341 |
| petG | cytochrome b6/f complex subunit V | Kenya_12_Local | MK455342 |
| petG | cytochrome b6/f complex subunit V | Kenya_13_Kibandameno | MK455343 |
| petG | cytochrome b6/f complex subunit V | Kenya_14_Kibandameno | MK455344 |
| petG | cytochrome b6/f complex subunit V | Kenya_15_Kibandameno | MK455345 |
| petG | cytochrome b6/f complex subunit V | Mozambique_4_Ezalamalithi | MK455346 |
| petG | cytochrome b6/f complex subunit V | Mozambique_5_Calamidade | MK455347 |
| petG | cytochrome b6/f complex subunit V | Mozambique_8_Calamidade | MK455348 |
| petG | cytochrome b6/f complex subunit V | Mozambique_10_Bwana | MK455349 |
| petG | cytochrome b6/f complex subunit V | Mozambique_11_Fernando | MK455350 |
| petG | cytochrome b6/f complex subunit V | Mozambique_16_Bwana | MK455351 |
| petG | cytochrome b6/f complex subunit V | Mozambique_17_Mulaleia | MK455352 |
| petG | cytochrome b6/f complex subunit V | Mozambique_20_Cadri | MK455353 |
| petG | cytochrome b6/f complex subunit V | Mozambique_23_Mulaleia | MK455354 |
| petG | cytochrome b6/f complex subunit V | Mozambique_Manihot_glaziovii | MK455355 |
| psaJ | photosystem I subunit IX | Tanzania_Healthy | MK455356 |
| psaJ | photosystem I subunit IX | Tanzania_03_Rumara | MK455357 |
| psaJ | photosystem I subunit IX | Tanzania_04_Kibandameno | MK455358 |
| psaJ | photosystem I subunit IX | Tanzania_05_Mkunungu | MK455359 |
| psaJ | photosystem I subunit IX | Tanzania_06_Kibandameno | MK455360 |
| psaJ | photosystem I subunit IX | Tanzania_07_Unknown | MK455361 |
| psaJ | photosystem I subunit IX | Tanzania_08_Unknown | MK455362 |
| psaJ | photosystem I subunit IX | Tanzania_09_Mkangawandu | MK455363 |
| psaJ | photosystem I subunit IX | Tanzania_10_Kibembe | MK455364 |
| psaJ | photosystem I subunit IX | Tanzania_11_Kibembe | MK455365 |
| psaJ | photosystem I subunit IX | Tanzania_12_Mwarabu | MK455366 |
| psaJ | photosystem I subunit IX | Kenya_01_Local | MK455367 |
| psaJ | photosystem I subunit IX | Kenya_02_Megana | MK455368 |
| psaJ | photosystem I subunit IX | Kenya_03_Tereka | MK455369 |
| psaJ | photosystem I subunit IX | Kenya_04_Megana | MK455370 |
| psaJ | photosystem I subunit IX | Kenya_05_Megana | MK455371 |
| psaJ | photosystem I subunit IX | Kenya_07_Local | MK455372 |
| psaJ | photosystem I subunit IX | Kenya_10_Local | MK455373 |
| psaJ | photosystem I subunit IX | Kenya_11_Kibandameno | MK455374 |
| psaJ | photosystem I subunit IX | Kenya_12_Local | MK455375 |
| psaJ | photosystem I subunit IX | Kenya_13_Kibandameno | MK455376 |
| psaJ | photosystem I subunit IX | Kenya_14_Kibandameno | MK455377 |
| psaJ | photosystem I subunit IX | Kenya_15_Kibandameno | MK455378 |
| psaJ | photosystem I subunit IX | Mozambique_4_Ezalamalithi | MK455379 |
| psaJ | photosystem I subunit IX | Mozambique_5_Calamidade | MK455380 |
| psaJ | photosystem I subunit IX | Mozambique_8_Calamidade | MK455381 |
| psaJ | photosystem I subunit IX | Mozambique_10_Bwana | MK455382 |
| psaJ | photosystem I subunit IX | Mozambique_11_Fernando | MK455383 |
| psaJ | photosystem I subunit IX | Mozambique_16_Bwana | MK455384 |
| psaJ | photosystem I subunit IX | Mozambique_17_Mulaleia | MK455385 |
| psaJ | photosystem I subunit IX | Mozambique_20_Cadri | MK455386 |
| psaJ | photosystem I subunit IX | Mozambique_23_Mulaleia | MK455387 |
| psaJ | photosystem I subunit IX | Mozambique_Manihot_glaziovii | MK455388 |
| rpl33 | ribosomal protein L33 | Tanzania_Healthy | MK455389 |
| rpl33 | ribosomal protein L33 | Tanzania_03_Rumara | MK455390 |
| rpl33 | ribosomal protein L33 | Tanzania_04_Kibandameno | MK455391 |
| rpl33 | ribosomal protein L33 | Tanzania_05_Mkunungu | MK455392 |
| rpl33 | ribosomal protein L33 | Tanzania_06_Kibandameno | MK455393 |
| rpl33 | ribosomal protein L33 | Tanzania_07_Unknown | MK455394 |
| rpl33 | ribosomal protein L33 | Tanzania_08_Unknown | MK455395 |
| rpl33 | ribosomal protein L33 | Tanzania_09_Mkangawandu | MK455396 |
| rpl33 | ribosomal protein L33 | Tanzania_10_Kibembe | MK455397 |
| rpl33 | ribosomal protein L33 | Tanzania_11_Kibembe | MK455398 |
| rpl33 | ribosomal protein L33 | Tanzania_12_Mwarabu | MK455399 |
| rpl33 | ribosomal protein L33 | Kenya_01_Local | MK455400 |
| rpl33 | ribosomal protein L33 | Kenya_02_Megana | MK455401 |
| rpl33 | ribosomal protein L33 | Kenya_03_Tereka | MK455402 |
| rpl33 | ribosomal protein L33 | Kenya_04_Megana | MK455403 |
| rpl33 | ribosomal protein L33 | Kenya_05_Megana | MK455404 |
| rpl33 | ribosomal protein L33 | Kenya_07_Local | MK455405 |
| rpl33 | ribosomal protein L33 | Kenya_10_Local | MK455406 |
| rpl33 | ribosomal protein L33 | Kenya_11_Kibandameno | MK455407 |
| rpl33 | ribosomal protein L33 | Kenya_12_Local | MK455408 |
| rpl33 | ribosomal protein L33 | Kenya_13_Kibandameno | MK455409 |
| rpl33 | ribosomal protein L33 | Kenya_14_Kibandameno | MK455410 |
| rpl33 | ribosomal protein L33 | Kenya_15_Kibandameno | MK455411 |
| rpl33 | ribosomal protein L33 | Mozambique_4_Ezalamalithi | MK455412 |
| rpl33 | ribosomal protein L33 | Mozambique_5_Calamidade | MK455413 |
| rpl33 | ribosomal protein L33 | Mozambique_8_Calamidade | MK455414 |
| rpl33 | ribosomal protein L33 | Mozambique_10_Bwana | MK455415 |
| rpl33 | ribosomal protein L33 | Mozambique_11_Fernando | MK455416 |
| rpl33 | ribosomal protein L33 | Mozambique_16_Bwana | MK455417 |
| rpl33 | ribosomal protein L33 | Mozambique_17_Mulaleia | MK455418 |
| rpl33 | ribosomal protein L33 | Mozambique_20_Cadri | MK455419 |
| rpl33 | ribosomal protein L33 | Mozambique_23_Mulaleia | MK455420 |
| rpl33 | ribosomal protein L33 | Mozambique_Manihot_glaziovii | MK455421 |
| rpl20 | ribosomal protein L20 | Tanzania_Healthy | MK455422 |
| rpl20 | ribosomal protein L20 | Tanzania_03_Rumara | MK455423 |
| rpl20 | ribosomal protein L20 | Tanzania_04_Kibandameno | MK455424 |
| rpl20 | ribosomal protein L20 | Tanzania_05_Mkunungu | MK455425 |
| rpl20 | ribosomal protein L20 | Tanzania_06_Kibandameno | MK455426 |
| rpl20 | ribosomal protein L20 | Tanzania_07_Unknown | MK455427 |
| rpl20 | ribosomal protein L20 | Tanzania_08_Unknown | MK455428 |
| rpl20 | ribosomal protein L20 | Tanzania_09_Mkangawandu | MK455429 |
| rpl20 | ribosomal protein L20 | Tanzania_10_Kibembe | MK455430 |
| rpl20 | ribosomal protein L20 | Tanzania_11_Kibembe | MK455431 |
| rpl20 | ribosomal protein L20 | Tanzania_12_Mwarabu | MK455432 |
| rpl20 | ribosomal protein L20 | Kenya_01_Local | MK455433 |
| rpl20 | ribosomal protein L20 | Kenya_02_Megana | MK455434 |
| rpl20 | ribosomal protein L20 | Kenya_03_Tereka | MK455435 |
| rpl20 | ribosomal protein L20 | Kenya_04_Megana | MK455436 |
| rpl20 | ribosomal protein L20 | Kenya_05_Megana | MK455437 |
| rpl20 | ribosomal protein L20 | Kenya_07_Local | MK455438 |
| rpl20 | ribosomal protein L20 | Kenya_10_Local | MK455439 |
| rpl20 | ribosomal protein L20 | Kenya_11_Kibandameno | MK455440 |
| rpl20 | ribosomal protein L20 | Kenya_12_Local | MK455441 |
| rpl20 | ribosomal protein L20 | Kenya_13_Kibandameno | MK455442 |
| rpl20 | ribosomal protein L20 | Kenya_14_Kibandameno | MK455443 |
| rpl20 | ribosomal protein L20 | Kenya_15_Kibandameno | MK455444 |
| rpl20 | ribosomal protein L20 | Mozambique_4_Ezalamalithi | MK455445 |
| rpl20 | ribosomal protein L20 | Mozambique_5_Calamidade | MK455446 |
| rpl20 | ribosomal protein L20 | Mozambique_8_Calamidade | MK455447 |
| rpl20 | ribosomal protein L20 | Mozambique_10_Bwana | MK455448 |
| rpl20 | ribosomal protein L20 | Mozambique_11_Fernando | MK455449 |
| rpl20 | ribosomal protein L20 | Mozambique_16_Bwana | MK455450 |
| rpl20 | ribosomal protein L20 | Mozambique_17_Mulaleia | MK455451 |
| rpl20 | ribosomal protein L20 | Mozambique_20_Cadri | MK455452 |
| rpl20 | ribosomal protein L20 | Mozambique_23_Mulaleia | MK455453 |
| rpl20 | ribosomal protein L20 | Mozambique_Manihot_glaziovii | MK455454 |
| clpP | clp protease proteolytic subunit | Tanzania_Healthy | MK455455 |
| clpP | clp protease proteolytic subunit | Tanzania_03_Rumara | MK455456 |
| clpP | clp protease proteolytic subunit | Tanzania_04_Kibandameno | MK455457 |
| clpP | clp protease proteolytic subunit | Tanzania_05_Mkunungu | MK455458 |
| clpP | clp protease proteolytic subunit | Tanzania_06_Kibandameno | MK455459 |
| clpP | clp protease proteolytic subunit | Tanzania_07_Unknown | MK455460 |
| clpP | clp protease proteolytic subunit | Tanzania_08_Unknown | MK455461 |
| clpP | clp protease proteolytic subunit | Tanzania_09_Mkangawandu | MK455462 |
| clpP | clp protease proteolytic subunit | Tanzania_10_Kibembe | MK455463 |
| clpP | clp protease proteolytic subunit | Tanzania_11_Kibembe | MK455464 |
| clpP | clp protease proteolytic subunit | Tanzania_12_Mwarabu | MK455465 |
| clpP | clp protease proteolytic subunit | Kenya_01_Local | MK455466 |
| clpP | clp protease proteolytic subunit | Kenya_02_Megana | MK455467 |
| clpP | clp protease proteolytic subunit | Kenya_03_Tereka | MK455468 |
| clpP | clp protease proteolytic subunit | Kenya_04_Megana | MK455469 |
| clpP | clp protease proteolytic subunit | Kenya_05_Megana | MK455470 |
| clpP | clp protease proteolytic subunit | Kenya_07_Local | MK455471 |
| clpP | clp protease proteolytic subunit | Kenya_10_Local | MK455472 |
| clpP | clp protease proteolytic subunit | Kenya_11_Kibandameno | MK455473 |
| clpP | clp protease proteolytic subunit | Kenya_12_Local | MK455474 |
| clpP | clp protease proteolytic subunit | Kenya_13_Kibandameno | MK455475 |
| clpP | clp protease proteolytic subunit | Kenya_14_Kibandameno | MK455476 |
| clpP | clp protease proteolytic subunit | Kenya_15_Kibandameno | MK455477 |
| clpP | clp protease proteolytic subunit | Mozambique_4_Ezalamalithi | MK455478 |
| clpP | clp protease proteolytic subunit | Mozambique_5_Calamidade | MK455479 |
| clpP | clp protease proteolytic subunit | Mozambique_8_Calamidade | MK455480 |
| clpP | clp protease proteolytic subunit | Mozambique_10_Bwana | MK455481 |
| clpP | clp protease proteolytic subunit | Mozambique_11_Fernando | MK455482 |
| clpP | clp protease proteolytic subunit | Mozambique_16_Bwana | MK455483 |
| clpP | clp protease proteolytic subunit | Mozambique_17_Mulaleia | MK455484 |
| clpP | clp protease proteolytic subunit | Mozambique_20_Cadri | MK455485 |
| clpP | clp protease proteolytic subunit | Mozambique_23_Mulaleia | MK455486 |
| clpP | clp protease proteolytic subunit | Mozambique_Manihot_glaziovii | MK455487 |
| psbB | photosystem II CP47 chlorophyll apoprotein | Tanzania_Healthy | MK455488 |
| psbB | photosystem II CP47 chlorophyll apoprotein | Tanzania_03_Rumara | MK455489 |
| psbB | photosystem II CP47 chlorophyll apoprotein | Tanzania_04_Kibandameno | MK455490 |
| psbB | photosystem II CP47 chlorophyll apoprotein | Tanzania_05_Mkunungu | MK455491 |
| psbB | photosystem II CP47 chlorophyll apoprotein | Tanzania_06_Kibandameno | MK455492 |
| psbB | photosystem II CP47 chlorophyll apoprotein | Tanzania_07_Unknown | MK455493 |
| psbB | photosystem II CP47 chlorophyll apoprotein | Tanzania_08_Unknown | MK455494 |
| psbB | photosystem II CP47 chlorophyll apoprotein | Tanzania_09_Mkangawandu | MK455495 |
| psbB | photosystem II CP47 chlorophyll apoprotein | Tanzania_10_Kibembe | MK455496 |
| psbB | photosystem II CP47 chlorophyll apoprotein | Tanzania_11_Kibembe | MK455497 |
| psbB | photosystem II CP47 chlorophyll apoprotein | Tanzania_12_Mwarabu | MK455498 |
| psbB | photosystem II CP47 chlorophyll apoprotein | Kenya_01_Local | MK455499 |
| psbB | photosystem II CP47 chlorophyll apoprotein | Kenya_02_Megana | MK455500 |
| psbB | photosystem II CP47 chlorophyll apoprotein | Kenya_03_Tereka | MK455501 |
| psbB | photosystem II CP47 chlorophyll apoprotein | Kenya_04_Megana | MK455502 |
| psbB | photosystem II CP47 chlorophyll apoprotein | Kenya_05_Megana | MK455503 |
| psbB | photosystem II CP47 chlorophyll apoprotein | Kenya_07_Local | MK455504 |
| psbB | photosystem II CP47 chlorophyll apoprotein | Kenya_10_Local | MK455505 |
| psbB | photosystem II CP47 chlorophyll apoprotein | Kenya_11_Kibandameno | MK455506 |
| psbB | photosystem II CP47 chlorophyll apoprotein | Kenya_12_Local | MK455507 |
| psbB | photosystem II CP47 chlorophyll apoprotein | Kenya_13_Kibandameno | MK455508 |
| psbB | photosystem II CP47 chlorophyll apoprotein | Kenya_14_Kibandameno | MK455509 |
| psbB | photosystem II CP47 chlorophyll apoprotein | Kenya_15_Kibandameno | MK455510 |
| psbB | photosystem II CP47 chlorophyll apoprotein | Mozambique_4_Ezalamalithi | MK455511 |
| psbB | photosystem II CP47 chlorophyll apoprotein | Mozambique_5_Calamidade | MK455512 |
| psbB | photosystem II CP47 chlorophyll apoprotein | Mozambique_8_Calamidade | MK455513 |
| psbB | photosystem II CP47 chlorophyll apoprotein | Mozambique_10_Bwana | MK455514 |
| psbB | photosystem II CP47 chlorophyll apoprotein | Mozambique_11_Fernando | MK455515 |
| psbB | photosystem II CP47 chlorophyll apoprotein | Mozambique_16_Bwana | MK455516 |
| psbB | photosystem II CP47 chlorophyll apoprotein | Mozambique_17_Mulaleia | MK455517 |
| psbB | photosystem II CP47 chlorophyll apoprotein | Mozambique_20_Cadri | MK455518 |
| psbB | photosystem II CP47 chlorophyll apoprotein | Mozambique_23_Mulaleia | MK455519 |
| psbB | photosystem II CP47 chlorophyll apoprotein | Mozambique_Manihot_glaziovii | MK455520 |
| psbT | photosystem II protein T | Tanzania_Healthy | MK455521 |
| psbT | photosystem II protein T | Tanzania_03_Rumara | MK455522 |
| psbT | photosystem II protein T | Tanzania_04_Kibandameno | MK455523 |
| psbT | photosystem II protein T | Tanzania_05_Mkunungu | MK455524 |
| psbT | photosystem II protein T | Tanzania_06_Kibandameno | MK455525 |
| psbT | photosystem II protein T | Tanzania_07_Unknown | MK455526 |
| psbT | photosystem II protein T | Tanzania_08_Unknown | MK455527 |
| psbT | photosystem II protein T | Tanzania_09_Mkangawandu | MK455528 |
| psbT | photosystem II protein T | Tanzania_10_Kibembe | MK455529 |
| psbT | photosystem II protein T | Tanzania_11_Kibembe | MK455530 |
| psbT | photosystem II protein T | Tanzania_12_Mwarabu | MK455531 |
| psbT | photosystem II protein T | Kenya_01_Local | MK455532 |
| psbT | photosystem II protein T | Kenya_02_Megana | MK455533 |
| psbT | photosystem II protein T | Kenya_03_Tereka | MK455534 |
| psbT | photosystem II protein T | Kenya_04_Megana | MK455535 |
| psbT | photosystem II protein T | Kenya_05_Megana | MK455536 |
| psbT | photosystem II protein T | Kenya_07_Local | MK455537 |
| psbT | photosystem II protein T | Kenya_10_Local | MK455538 |
| psbT | photosystem II protein T | Kenya_11_Kibandameno | MK455539 |
| psbT | photosystem II protein T | Kenya_12_Local | MK455540 |
| psbT | photosystem II protein T | Kenya_13_Kibandameno | MK455541 |
| psbT | photosystem II protein T | Kenya_14_Kibandameno | MK455542 |
| psbT | photosystem II protein T | Kenya_15_Kibandameno | MK455543 |
| psbT | photosystem II protein T | Mozambique_4_Ezalamalithi | MK455544 |
| psbT | photosystem II protein T | Mozambique_5_Calamidade | MK455545 |
| psbT | photosystem II protein T | Mozambique_8_Calamidade | MK455546 |
| psbT | photosystem II protein T | Mozambique_10_Bwana | MK455547 |
| psbT | photosystem II protein T | Mozambique_11_Fernando | MK455548 |
| psbT | photosystem II protein T | Mozambique_16_Bwana | MK455549 |
| psbT | photosystem II protein T | Mozambique_17_Mulaleia | MK455550 |
| psbT | photosystem II protein T | Mozambique_20_Cadri | MK455551 |
| psbT | photosystem II protein T | Mozambique_23_Mulaleia | MK455552 |
| psbT | photosystem II protein T | Mozambique_Manihot_glaziovii | MK455553 |
| psbN | photosystem II protein N | Tanzania_Healthy | MK455554 |
| psbN | photosystem II protein N | Tanzania_03_Rumara | MK455555 |
| psbN | photosystem II protein N | Tanzania_04_Kibandameno | MK455556 |
| psbN | photosystem II protein N | Tanzania_05_Mkunungu | MK455557 |
| psbN | photosystem II protein N | Tanzania_06_Kibandameno | MK455558 |
| psbN | photosystem II protein N | Tanzania_07_Unknown | MK455559 |
| psbN | photosystem II protein N | Tanzania_08_Unknown | MK455560 |
| psbN | photosystem II protein N | Tanzania_09_Mkangawandu | MK455561 |
| psbN | photosystem II protein N | Tanzania_10_Kibembe | MK455562 |
| psbN | photosystem II protein N | Tanzania_11_Kibembe | MK455563 |
| psbN | photosystem II protein N | Tanzania_12_Mwarabu | MK455564 |
| psbN | photosystem II protein N | Kenya_01_Local | MK455565 |
| psbN | photosystem II protein N | Kenya_02_Megana | MK455566 |
| psbN | photosystem II protein N | Kenya_03_Tereka | MK455567 |
| psbN | photosystem II protein N | Kenya_04_Megana | MK455568 |
| psbN | photosystem II protein N | Kenya_05_Megana | MK455569 |
| psbN | photosystem II protein N | Kenya_07_Local | MK455570 |
| psbN | photosystem II protein N | Kenya_10_Local | MK455571 |
| psbN | photosystem II protein N | Kenya_11_Kibandameno | MK455572 |
| psbN | photosystem II protein N | Kenya_12_Local | MK455573 |
| psbN | photosystem II protein N | Kenya_13_Kibandameno | MK455574 |
| psbN | photosystem II protein N | Kenya_14_Kibandameno | MK455575 |
| psbN | photosystem II protein N | Kenya_15_Kibandameno | MK455576 |
| psbN | photosystem II protein N | Mozambique_4_Ezalamalithi | MK455577 |
| psbN | photosystem II protein N | Mozambique_5_Calamidade | MK455578 |
| psbN | photosystem II protein N | Mozambique_8_Calamidade | MK455579 |
| psbN | photosystem II protein N | Mozambique_10_Bwana | MK455580 |
| psbN | photosystem II protein N | Mozambique_11_Fernando | MK455581 |
| psbN | photosystem II protein N | Mozambique_16_Bwana | MK455582 |
| psbN | photosystem II protein N | Mozambique_17_Mulaleia | MK455583 |
| psbN | photosystem II protein N | Mozambique_20_Cadri | MK455584 |
| psbN | photosystem II protein N | Mozambique_23_Mulaleia | MK455585 |
| psbN | photosystem II protein N | Mozambique_Manihot_glaziovii | MK455586 |
| psbH | photosystem II phosphoprotein | Tanzania_Healthy | MK455587 |
| psbH | photosystem II phosphoprotein | Tanzania_03_Rumara | MK455588 |
| psbH | photosystem II phosphoprotein | Tanzania_04_Kibandameno | MK455589 |
| psbH | photosystem II phosphoprotein | Tanzania_05_Mkunungu | MK455590 |
| psbH | photosystem II phosphoprotein | Tanzania_06_Kibandameno | MK455591 |
| psbH | photosystem II phosphoprotein | Tanzania_07_Unknown | MK455592 |
| psbH | photosystem II phosphoprotein | Tanzania_08_Unknown | MK455593 |
| psbH | photosystem II phosphoprotein | Tanzania_09_Mkangawandu | MK455594 |
| psbH | photosystem II phosphoprotein | Tanzania_10_Kibembe | MK455595 |
| psbH | photosystem II phosphoprotein | Tanzania_11_Kibembe | MK455596 |
| psbH | photosystem II phosphoprotein | Tanzania_12_Mwarabu | MK455597 |
| psbH | photosystem II phosphoprotein | Kenya_01_Local | MK455598 |
| psbH | photosystem II phosphoprotein | Kenya_02_Megana | MK455599 |
| psbH | photosystem II phosphoprotein | Kenya_03_Tereka | MK455600 |
| psbH | photosystem II phosphoprotein | Kenya_04_Megana | MK455601 |
| psbH | photosystem II phosphoprotein | Kenya_05_Megana | MK455602 |
| psbH | photosystem II phosphoprotein | Kenya_07_Local | MK455603 |
| psbH | photosystem II phosphoprotein | Kenya_10_Local | MK455604 |
| psbH | photosystem II phosphoprotein | Kenya_11_Kibandameno | MK455605 |
| psbH | photosystem II phosphoprotein | Kenya_12_Local | MK455606 |
| psbH | photosystem II phosphoprotein | Kenya_13_Kibandameno | MK455607 |
| psbH | photosystem II phosphoprotein | Kenya_14_Kibandameno | MK455608 |
| psbH | photosystem II phosphoprotein | Kenya_15_Kibandameno | MK455609 |
| psbH | photosystem II phosphoprotein | Mozambique_4_Ezalamalithi | MK455610 |
| psbH | photosystem II phosphoprotein | Mozambique_5_Calamidade | MK455611 |
| psbH | photosystem II phosphoprotein | Mozambique_8_Calamidade | MK455612 |
| psbH | photosystem II phosphoprotein | Mozambique_10_Bwana | MK455613 |
| psbH | photosystem II phosphoprotein | Mozambique_11_Fernando | MK455614 |
| psbH | photosystem II phosphoprotein | Mozambique_16_Bwana | MK455615 |
| psbH | photosystem II phosphoprotein | Mozambique_17_Mulaleia | MK455616 |
| psbH | photosystem II phosphoprotein | Mozambique_20_Cadri | MK455617 |
| psbH | photosystem II phosphoprotein | Mozambique_23_Mulaleia | MK455618 |
| psbH | photosystem II phosphoprotein | Mozambique_Manihot_glaziovi | MK455619 |
| petB | cytochrome b6 | Tanzania_Healthy | MK455620 |
| petB | cytochrome b6 | Tanzania_03_Rumara | MK455621 |
| petB | cytochrome b6 | Tanzania_04_Kibandameno | MK455622 |
| petB | cytochrome b6 | Tanzania_05_Mkunungu | MK455623 |
| petB | cytochrome b6 | Tanzania_06_Kibandameno | MK455624 |
| petB | cytochrome b6 | Tanzania_07_Unknown | MK455625 |
| petB | cytochrome b6 | Tanzania_08_Unknown | MK455626 |
| petB | cytochrome b6 | Tanzania_09_Mkangawandu | MK455627 |
| petB | cytochrome b6 | Tanzania_10_Kibembe | MK455628 |
| petB | cytochrome b6 | Tanzania_11_Kibembe | MK455629 |
| petB | cytochrome b6 | Tanzania_12_Mwarabu | MK455630 |
| petB | cytochrome b6 | Kenya_01_Local | MK455631 |
| petB | cytochrome b6 | Kenya_02_Megana | MK455632 |
| petB | cytochrome b6 | Kenya_03_Tereka | MK455633 |
| petB | cytochrome b6 | Kenya_04_Megana | MK455634 |
| petB | cytochrome b6 | Kenya_05_Megana | MK455635 |
| petB | cytochrome b6 | Kenya_07_Local | MK455636 |
| petB | cytochrome b6 | Kenya_10_Local | MK455637 |
| petB | cytochrome b6 | Kenya_11_Kibandameno | MK455638 |
| petB | cytochrome b6 | Kenya_12_Local | MK455639 |
| petB | cytochrome b6 | Kenya_13_Kibandameno | MK455640 |
| petB | cytochrome b6 | Kenya_14_Kibandameno | MK455641 |
| petB | cytochrome b6 | Kenya_15_Kibandameno | MK455642 |
| petB | cytochrome b6 | Mozambique_4_Ezalamalithi | MK455643 |
| petB | cytochrome b6 | Mozambique_5_Calamidade | MK455644 |
| petB | cytochrome b6 | Mozambique_8_Calamidade | MK455645 |
| petB | cytochrome b6 | Mozambique_10_Bwana | MK455646 |
| petB | cytochrome b6 | Mozambique_11_Fernando | MK455647 |
| petB | cytochrome b6 | Mozambique_16_Bwana | MK455648 |
| petB | cytochrome b6 | Mozambique_17_Mulaleia | MK455649 |
| petB | cytochrome b6 | Mozambique_20_Cadri | MK455650 |
| petB | cytochrome b6 | Mozambique_23_Mulaleia | MK455651 |
| petB | cytochrome b6 | Mozambique_Manihot_glaziovii | MK455652 |
| rps11 | ribosomal protein S11 | Tanzania_Healthy | MK455653 |
| rps11 | ribosomal protein S11 | Tanzania_03_Rumara | MK455654 |
| rps11 | ribosomal protein S11 | Tanzania_04_Kibandameno | MK455655 |
| rps11 | ribosomal protein S11 | Tanzania_05_Mkunungu | MK455656 |
| rps11 | ribosomal protein S11 | Tanzania_06_Kibandameno | MK455657 |
| rps11 | ribosomal protein S11 | Tanzania_07_Unknown | MK455658 |
| rps11 | ribosomal protein S11 | Tanzania_08_Unknown | MK455659 |
| rps11 | ribosomal protein S11 | Tanzania_09_Mkangawandu | MK455660 |
| rps11 | ribosomal protein S11 | Tanzania_10_Kibembe | MK455661 |
| rps11 | ribosomal protein S11 | Tanzania_11_Kibembe | MK455662 |
| rps11 | ribosomal protein S11 | Tanzania_12_Mwarabu | MK455663 |
| rps11 | ribosomal protein S11 | Kenya_01_Local | MK455664 |
| rps11 | ribosomal protein S11 | Kenya_02_Megana | MK455665 |
| rps11 | ribosomal protein S11 | Kenya_03_Tereka | MK455666 |
| rps11 | ribosomal protein S11 | Kenya_04_Megana | MK455667 |
| rps11 | ribosomal protein S11 | Kenya_05_Megana | MK455668 |
| rps11 | ribosomal protein S11 | Kenya_07_Local | MK455669 |
| rps11 | ribosomal protein S11 | Kenya_10_Local | MK455670 |
| rps11 | ribosomal protein S11 | Kenya_11_Kibandameno | MK455671 |
| rps11 | ribosomal protein S11 | Kenya_12_Local | MK455672 |
| rps11 | ribosomal protein S11 | Kenya_13_Kibandameno | MK455673 |
| rps11 | ribosomal protein S11 | Kenya_14_Kibandameno | MK455674 |
| rps11 | ribosomal protein S11 | Kenya_15_Kibandameno | MK455675 |
| rps11 | ribosomal protein S11 | Mozambique_4_Ezalamalithi | MK455676 |
| rps11 | ribosomal protein S11 | Mozambique_5_Calamidade | MK455677 |
| rps11 | ribosomal protein S11 | Mozambique_8_Calamidade | MK455678 |
| rps11 | ribosomal protein S11 | Mozambique_10_Bwana | MK455679 |
| rps11 | ribosomal protein S11 | Mozambique_11_Fernando | MK455680 |
| rps11 | ribosomal protein S11 | Mozambique_16_Bwana | MK455681 |
| rps11 | ribosomal protein S11 | Mozambique_17_Mulaleia | MK455682 |
| rps11 | ribosomal protein S11 | Mozambique_20_Cadri | MK455683 |
| rps11 | ribosomal protein S11 | Mozambique_23_Mulaleia | MK455684 |
| rps11 | ribosomal protein S11 | Mozambique_Manihot_glaziovii | MK455685 |
| rpl36 | ribosomal protein L36 | Tanzania_Healthy | MK455686 |
| rpl36 | ribosomal protein L36 | Tanzania_03_Rumara | MK455687 |
| rpl36 | ribosomal protein L36 | Tanzania_04_Kibandameno | MK455688 |
| rpl36 | ribosomal protein L36 | Tanzania_05_Mkunungu | MK455689 |
| rpl36 | ribosomal protein L36 | Tanzania_06_Kibandameno | MK455690 |
| rpl36 | ribosomal protein L36 | Tanzania_07_Unknown | MK455691 |
| rpl36 | ribosomal protein L36 | Tanzania_08_Unknown | MK455692 |
| rpl36 | ribosomal protein L36 | Tanzania_09_Mkangawandu | MK455693 |
| rpl36 | ribosomal protein L36 | Tanzania_10_Kibembe | MK455694 |
| rpl36 | ribosomal protein L36 | Tanzania_11_Kibembe | MK455695 |
| rpl36 | ribosomal protein L36 | Tanzania_12_Mwarabu | MK455696 |
| rpl36 | ribosomal protein L36 | Kenya_01_Local | MK455697 |
| rpl36 | ribosomal protein L36 | Kenya_02_Megana | MK455698 |
| rpl36 | ribosomal protein L36 | Kenya_03_Tereka | MK455699 |
| rpl36 | ribosomal protein L36 | Kenya_04_Megana | MK455700 |
| rpl36 | ribosomal protein L36 | Kenya_05_Megana | MK455701 |
| rpl36 | ribosomal protein L36 | Kenya_07_Local | MK455702 |
| rpl36 | ribosomal protein L36 | Kenya_10_Local | MK455703 |
| rpl36 | ribosomal protein L36 | Kenya_11_Kibandameno | MK455704 |
| rpl36 | ribosomal protein L36 | Kenya_12_Local | MK455705 |
| rpl36 | ribosomal protein L36 | Kenya_13_Kibandameno | MK455706 |
| rpl36 | ribosomal protein L36 | Kenya_14_Kibandameno | MK455707 |
| rpl36 | ribosomal protein L36 | Kenya_15_Kibandameno | MK455708 |
| rpl36 | ribosomal protein L36 | Mozambique_4_Ezalamalithi | MK455709 |
| rpl36 | ribosomal protein L36 | Mozambique_5_Calamidade | MK455710 |
| rpl36 | ribosomal protein L36 | Mozambique_8_Calamidade | MK455711 |
| rpl36 | ribosomal protein L36 | Mozambique_10_Bwana | MK455712 |
| rpl36 | ribosomal protein L36 | Mozambique_11_Fernando | MK455713 |
| rpl36 | ribosomal protein L36 | Mozambique_16_Bwana | MK455714 |
| rpl36 | ribosomal protein L36 | Mozambique_17_Mulaleia | MK455715 |
| rpl36 | ribosomal protein L36 | Mozambique_20_Cadri | MK455716 |
| rpl36 | ribosomal protein L36 | Mozambique_23_Mulaleia | MK455717 |
| rpl36 | ribosomal protein L36 | Mozambique_Manihot_glaziovii | MK455718 |
| rps8 | ribosomal protein S8 | Tanzania_Healthy | MK455719 |
| rps8 | ribosomal protein S8 | Tanzania_03_Rumara | MK455720 |
| rps8 | ribosomal protein S8 | Tanzania_04_Kibandameno | MK455721 |
| rps8 | ribosomal protein S8 | Tanzania_05_Mkunungu | MK455722 |
| rps8 | ribosomal protein S8 | Tanzania_06_Kibandameno | MK455723 |
| rps8 | ribosomal protein S8 | Tanzania_07_Unknown | MK455724 |
| rps8 | ribosomal protein S8 | Tanzania_08_Unknown | MK455725 |
| rps8 | ribosomal protein S8 | Tanzania_09_Mkangawandu | MK455726 |
| rps8 | ribosomal protein S8 | Tanzania_10_Kibembe | MK455727 |
| rps8 | ribosomal protein S8 | Tanzania_11_Kibembe | MK455728 |
| rps8 | ribosomal protein S8 | Tanzania_12_Mwarabu | MK455729 |
| rps8 | ribosomal protein S8 | Kenya_01_Local | MK455730 |
| rps8 | ribosomal protein S8 | Kenya_02_Megana | MK455731 |
| rps8 | ribosomal protein S8 | Kenya_03_Tereka | MK455732 |
| rps8 | ribosomal protein S8 | Kenya_04_Megana | MK455733 |
| rps8 | ribosomal protein S8 | Kenya_05_Megana | MK455734 |
| rps8 | ribosomal protein S8 | Kenya_07_Local | MK455735 |
| rps8 | ribosomal protein S8 | Kenya_10_Local | MK455736 |
| rps8 | ribosomal protein S8 | Kenya_11_Kibandameno | MK455737 |
| rps8 | ribosomal protein S8 | Kenya_12_Local | MK455738 |
| rps8 | ribosomal protein S8 | Kenya_13_Kibandameno | MK455739 |
| rps8 | ribosomal protein S8 | Kenya_14_Kibandameno | MK455740 |
| rps8 | ribosomal protein S8 | Kenya_15_Kibandameno | MK455741 |
| rps8 | ribosomal protein S8 | Mozambique_4_Ezalamalithi | MK455742 |
| rps8 | ribosomal protein S8 | Mozambique_5_Calamidade | MK455743 |
| rps8 | ribosomal protein S8 | Mozambique_8_Calamidade | MK455744 |
| rps8 | ribosomal protein S8 | Mozambique_10_Bwana | MK455745 |
| rps8 | ribosomal protein S8 | Mozambique_11_Fernando | MK455746 |
| rps8 | ribosomal protein S8 | Mozambique_16_Bwana | MK455747 |
| rps8 | ribosomal protein S8 | Mozambique_17_Mulaleia | MK455748 |
| rps8 | ribosomal protein S8 | Mozambique_20_Cadri | MK455749 |
| rps8 | ribosomal protein S8 | Mozambique_23_Mulaleia | MK455750 |
| rps8 | ribosomal protein S8 | Mozambique_Manihot_glaziovii | MK455751 |
| rpl14 | ribosomal protein L14 | Tanzania_Healthy | MK470119 |
| rpl14 | ribosomal protein L14 | Tanzania_03_Rumara | MK470120 |
| rpl14 | ribosomal protein L14 | Tanzania_04_Kibandameno | MK470121 |
| rpl14 | ribosomal protein L14 | Tanzania_05_Mkunungu | MK470122 |
| rpl14 | ribosomal protein L14 | Tanzania_06_Kibandameno | MK470123 |
| rpl14 | ribosomal protein L14 | Tanzania_07_Unknown | MK470124 |
| rpl14 | ribosomal protein L14 | Tanzania_08_Unknown | MK470125 |
| rpl14 | ribosomal protein L14 | Tanzania_09_Mkangawandu | MK470126 |
| rpl14 | ribosomal protein L14 | Tanzania_10_Kibembe | MK470127 |
| rpl14 | ribosomal protein L14 | Tanzania_11_Kibembe | MK470128 |
| rpl14 | ribosomal protein L14 | Tanzania_12_Mwarabu | MK470129 |
| rpl14 | ribosomal protein L14 | Kenya_01_Local | MK470130 |
| rpl14 | ribosomal protein L14 | Kenya_02_Megana | MK470131 |
| rpl14 | ribosomal protein L14 | Kenya_03_Tereka | MK470132 |
| rpl14 | ribosomal protein L14 | Kenya_04_Megana | MK470133 |
| rpl14 | ribosomal protein L14 | Kenya_05_Megana | MK470134 |
| rpl14 | ribosomal protein L14 | Kenya_07_Local | MK470135 |
| rpl14 | ribosomal protein L14 | Kenya_10_Local | MK470136 |
| rpl14 | ribosomal protein L14 | Kenya_11_Kibandameno | MK470137 |
| rpl14 | ribosomal protein L14 | Kenya_12_Local | MK470138 |
| rpl14 | ribosomal protein L14 | Kenya_13_Kibandameno | MK470139 |
| rpl14 | ribosomal protein L14 | Kenya_14_Kibandameno | MK470140 |
| rpl14 | ribosomal protein L14 | Kenya_15_Kibandameno | MK470141 |
| rpl14 | ribosomal protein L14 | Mozambique_4_Ezalamalithi | MK470142 |
| rpl14 | ribosomal protein L14 | Mozambique_5_Calamidade | MK470143 |
| rpl14 | ribosomal protein L14 | Mozambique_8_Calamidade | MK470144 |
| rpl14 | ribosomal protein L14 | Mozambique_10_Bwana | MK470145 |
| rpl14 | ribosomal protein L14 | Mozambique_11_Fernando | MK470146 |
| rpl14 | ribosomal protein L14 | Mozambique_16_Bwana | MK470147 |
| rpl14 | ribosomal protein L14 | Mozambique_17_Mulaleia | MK470148 |
| rpl14 | ribosomal protein L14 | Mozambique_20_Cadri | MK470149 |
| rpl14 | ribosomal protein L14 | Mozambique_23_Mulaleia | MK470150 |
| rpl14 | ribosomal protein L14 | Mozambique_Manihot_glaziovii | MK470151 |
| rpl16 | ribosomal protein L16 | Tanzania_Healthy | MK470152 |
| rpl16 | ribosomal protein L16 | Tanzania_03_Rumara | MK470153 |
| rpl16 | ribosomal protein L16 | Tanzania_04_Kibandameno | MK470154 |
| rpl16 | ribosomal protein L16 | Tanzania_05_Mkunungu | MK470155 |
| rpl16 | ribosomal protein L16 | Tanzania_06_Kibandameno | MK470156 |
| rpl16 | ribosomal protein L16 | Tanzania_07_Unknown | MK470157 |
| rpl16 | ribosomal protein L16 | Tanzania_08_Unknown | MK470158 |
| rpl16 | ribosomal protein L16 | Tanzania_09_Mkangawandu | MK470159 |
| rpl16 | ribosomal protein L16 | Tanzania_10_Kibembe | MK470160 |
| rpl16 | ribosomal protein L16 | Tanzania_11_Kibembe | MK470161 |
| rpl16 | ribosomal protein L16 | Tanzania_12_Mwarabu | MK470162 |
| rpl16 | ribosomal protein L16 | Kenya_01_Local | MK470163 |
| rpl16 | ribosomal protein L16 | Kenya_02_Megana | MK470164 |
| rpl16 | ribosomal protein L16 | Kenya_03_Tereka | MK470165 |
| rpl16 | ribosomal protein L16 | Kenya_04_Megana | MK470166 |
| rpl16 | ribosomal protein L16 | Kenya_05_Megana | MK470167 |
| rpl16 | ribosomal protein L16 | Kenya_07_Local | MK470168 |
| rpl16 | ribosomal protein L16 | Kenya_10_Local | MK470169 |
| rpl16 | ribosomal protein L16 | Kenya_11_Kibandameno | MK470170 |
| rpl16 | ribosomal protein L16 | Kenya_12_Local | MK470171 |
| rpl16 | ribosomal protein L16 | Kenya_13_Kibandameno | MK470172 |
| rpl16 | ribosomal protein L16 | Kenya_14_Kibandameno | MK470173 |
| rpl16 | ribosomal protein L16 | Kenya_15_Kibandameno | MK470174 |
| rpl16 | ribosomal protein L16 | Mozambique_4_Ezalamalithi | MK470175 |
| rpl16 | ribosomal protein L16 | Mozambique_5_Calamidade | MK470176 |
| rpl16 | ribosomal protein L16 | Mozambique_8_Calamidade | MK470177 |
| rpl16 | ribosomal protein L16 | Mozambique_10_Bwana | MK470178 |
| rpl16 | ribosomal protein L16 | Mozambique_11_Fernando | MK470179 |
| rpl16 | ribosomal protein L16 | Mozambique_16_Bwana | MK470180 |
| rpl16 | ribosomal protein L16 | Mozambique_17_Mulaleia | MK470181 |
| rpl16 | ribosomal protein L16 | Mozambique_20_Cadri | MK470182 |
| rpl16 | ribosomal protein L16 | Mozambique_23_Mulaleia | MK470183 |
| rpl16 | ribosomal protein L16 | Mozambique_Manihot_glaziovii | MK470184 |
| rps3 | ribosomal protein S3 | Tanzania_Healthy | MK470185 |
| rps3 | ribosomal protein S3 | Tanzania_03_Rumara | MK470186 |
| rps3 | ribosomal protein S3 | Tanzania_04_Kibandameno | MK470187 |
| rps3 | ribosomal protein S3 | Tanzania_05_Mkunungu | MK470188 |
| rps3 | ribosomal protein S3 | Tanzania_06_Kibandameno | MK470189 |
| rps3 | ribosomal protein S3 | Tanzania_07_Unknown | MK470190 |
| rps3 | ribosomal protein S3 | Tanzania_08_Unknown | MK470191 |
| rps3 | ribosomal protein S3 | Tanzania_09_Mkangawandu | MK470192 |
| rps3 | ribosomal protein S3 | Tanzania_10_Kibembe | MK470193 |
| rps3 | ribosomal protein S3 | Tanzania_11_Kibembe | MK470194 |
| rps3 | ribosomal protein S3 | Tanzania_12_Mwarabu | MK470195 |
| rps3 | ribosomal protein S3 | Kenya_01_Local | MK470196 |
| rps3 | ribosomal protein S3 | Kenya_02_Megana | MK470197 |
| rps3 | ribosomal protein S3 | Kenya_03_Tereka | MK470198 |
| rps3 | ribosomal protein S3 | Kenya_04_Megana | MK470199 |
| rps3 | ribosomal protein S3 | Kenya_05_Megana | MK470200 |
| rps3 | ribosomal protein S3 | Kenya_07_Local | MK470201 |
| rps3 | ribosomal protein S3 | Kenya_10_Local | MK470202 |
| rps3 | ribosomal protein S3 | Kenya_11_Kibandameno | MK470203 |
| rps3 | ribosomal protein S3 | Kenya_12_Local | MK470204 |
| rps3 | ribosomal protein S3 | Kenya_13_Kibandameno | MK470205 |
| rps3 | ribosomal protein S3 | Kenya_14_Kibandameno | MK470206 |
| rps3 | ribosomal protein S3 | Kenya_15_Kibandameno | MK470207 |
| rps3 | ribosomal protein S3 | Mozambique_4_Ezalamalithi | MK470208 |
| rps3 | ribosomal protein S3 | Mozambique_5_Calamidade | MK470209 |
| rps3 | ribosomal protein S3 | Mozambique_8_Calamidade | MK470210 |
| rps3 | ribosomal protein S3 | Mozambique_10_Bwana | MK470211 |
| rps3 | ribosomal protein S3 | Mozambique_11_Fernando | MK470212 |
| rps3 | ribosomal protein S3 | Mozambique_16_Bwana | MK470213 |
| rps3 | ribosomal protein S3 | Mozambique_17_Mulaleia | MK470214 |
| rps3 | ribosomal protein S3 | Mozambique_20_Cadri | MK470215 |
| rps3 | ribosomal protein S3 | Mozambique_23_Mulaleia | MK470216 |
| rps3 | ribosomal protein S3 | Mozambique_Manihot_glaziovii | MK470217 |
| rpl22 | ribosomal protein L22 | Tanzania_Healthy | MK470218 |
| rpl22 | ribosomal protein L22 | Tanzania_03_Rumara | MK470219 |
| rpl22 | ribosomal protein L22 | Tanzania_04_Kibandameno | MK470220 |
| rpl22 | ribosomal protein L22 | Tanzania_05_Mkunungu | MK470221 |
| rpl22 | ribosomal protein L22 | Tanzania_06_Kibandameno | MK470222 |
| rpl22 | ribosomal protein L22 | Tanzania_07_Unknown | MK470223 |
| rpl22 | ribosomal protein L22 | Tanzania_08_Unknown | MK470224 |
| rpl22 | ribosomal protein L22 | Tanzania_09_Mkangawandu | MK470225 |
| rpl22 | ribosomal protein L22 | Tanzania_10_Kibembe | MK470226 |
| rpl22 | ribosomal protein L22 | Tanzania_11_Kibembe | MK470227 |
| rpl22 | ribosomal protein L22 | Tanzania_12_Mwarabu | MK470228 |
| rpl22 | ribosomal protein L22 | Kenya_01_Local | MK470229 |
| rpl22 | ribosomal protein L22 | Kenya_02_Megana | MK470230 |
| rpl22 | ribosomal protein L22 | Kenya_03_Tereka | MK470231 |
| rpl22 | ribosomal protein L22 | Kenya_04_Megana | MK470232 |
| rpl22 | ribosomal protein L22 | Kenya_05_Megana | MK470233 |
| rpl22 | ribosomal protein L22 | Kenya_07_Local | MK470234 |
| rpl22 | ribosomal protein L22 | Kenya_10_Local | MK470235 |
| rpl22 | ribosomal protein L22 | Kenya_11_Kibandameno | MK470236 |
| rpl22 | ribosomal protein L22 | Kenya_12_Local | MK470237 |
| rpl22 | ribosomal protein L22 | Kenya_13_Kibandameno | MK470238 |
| rpl22 | ribosomal protein L22 | Kenya_14_Kibandameno | MK470239 |
| rpl22 | ribosomal protein L22 | Kenya_15_Kibandameno | MK470240 |
| rpl22 | ribosomal protein L22 | Mozambique_4_Ezalamalithi | MK470241 |
| rpl22 | ribosomal protein L22 | Mozambique_5_Calamidade | MK470242 |
| rpl22 | ribosomal protein L22 | Mozambique_8_Calamidade | MK470243 |
| rpl22 | ribosomal protein L22 | Mozambique_10_Bwana | MK470244 |
| rpl22 | ribosomal protein L22 | Mozambique_11_Fernando | MK470245 |
| rpl22 | ribosomal protein L22 | Mozambique_16_Bwana | MK470246 |
| rpl22 | ribosomal protein L22 | Mozambique_17_Mulaleia | MK470247 |
| rpl22 | ribosomal protein L22 | Mozambique_20_Cadri | MK470248 |
| rpl22 | ribosomal protein L22 | Mozambique_23_Mulaleia | MK470249 |
| rpl22 | ribosomal protein L22 | Mozambique_Manihot_glaziovii | MK470250 |
| rpl2 | ribosomal protein L2 | Tanzania_Healthy | MK470251 |
| rpl2 | ribosomal protein L2 | Tanzania_03_Rumara | MK470252 |
| rpl2 | ribosomal protein L2 | Tanzania_04_Kibandameno | MK470253 |
| rpl2 | ribosomal protein L2 | Tanzania_05_Mkunungu | MK470254 |
| rpl2 | ribosomal protein L2 | Tanzania_06_Kibandameno | MK470255 |
| rpl2 | ribosomal protein L2 | Tanzania_07_Unknown | MK470256 |
| rpl2 | ribosomal protein L2 | Tanzania_08_Unknown | MK470257 |
| rpl2 | ribosomal protein L2 | Tanzania_09_Mkangawandu | MK470258 |
| rpl2 | ribosomal protein L2 | Tanzania_10_Kibembe | MK470259 |
| rpl2 | ribosomal protein L2 | Tanzania_11_Kibembe | MK470260 |
| rpl2 | ribosomal protein L2 | Tanzania_12_Mwarabu | MK470261 |
| rpl2 | ribosomal protein L2 | Kenya_01_Local | MK470262 |
| rpl2 | ribosomal protein L2 | Kenya_02_Megana | MK470263 |
| rpl2 | ribosomal protein L2 | Kenya_03_Tereka | MK470264 |
| rpl2 | ribosomal protein L2 | Kenya_04_Megana | MK470265 |
| rpl2 | ribosomal protein L2 | Kenya_05_Megana | MK470266 |
| rpl2 | ribosomal protein L2 | Kenya_07_Local | MK470267 |
| rpl2 | ribosomal protein L2 | Kenya_10_Local | MK470268 |
| rpl2 | ribosomal protein L2 | Kenya_11_Kibandameno | MK470269 |
| rpl2 | ribosomal protein L2 | Kenya_12_Local | MK470270 |
| rpl2 | ribosomal protein L2 | Kenya_13_Kibandameno | MK470271 |
| rpl2 | ribosomal protein L2 | Kenya_14_Kibandameno | MK470272 |
| rpl2 | ribosomal protein L2 | Kenya_15_Kibandameno | MK470273 |
| rpl2 | ribosomal protein L2 | Mozambique_4_Ezalamalithi | MK470274 |
| rpl2 | ribosomal protein L2 | Mozambique_5_Calamidade | MK470275 |
| rpl2 | ribosomal protein L2 | Mozambique_8_Calamidade | MK470276 |
| rpl2 | ribosomal protein L2 | Mozambique_10_Bwana | MK470277 |
| rpl2 | ribosomal protein L2 | Mozambique_11_Fernando | MK470278 |
| rpl2 | ribosomal protein L2 | Mozambique_16_Bwana | MK470279 |
| rpl2 | ribosomal protein L2 | Mozambique_17_Mulaleia | MK470280 |
| rpl2 | ribosomal protein L2 | Mozambique_20_Cadri | MK470281 |
| rpl2 | ribosomal protein L2 | Mozambique_23_Mulaleia | MK470282 |
| rpl2 | ribosomal protein L2 | Mozambique_Manihot_glaziovii | MK470283 |
| rpl23 | ribosomal protein L23 | Tanzania_Healthy | MK470284 |
| rpl23 | ribosomal protein L23 | Tanzania_03_Rumara | MK470285 |
| rpl23 | ribosomal protein L23 | Tanzania_04_Kibandameno | MK470286 |
| rpl23 | ribosomal protein L23 | Tanzania_05_Mkunungu | MK470287 |
| rpl23 | ribosomal protein L23 | Tanzania_06_Kibandameno | MK470288 |
| rpl23 | ribosomal protein L23 | Tanzania_07_Unknown | MK470289 |
| rpl23 | ribosomal protein L23 | Tanzania_08_Unknown | MK470290 |
| rpl23 | ribosomal protein L23 | Tanzania_09_Mkangawandu | MK470291 |
| rpl23 | ribosomal protein L23 | Tanzania_10_Kibembe | MK470292 |
| rpl23 | ribosomal protein L23 | Tanzania_11_Kibembe | MK470293 |
| rpl23 | ribosomal protein L23 | Tanzania_12_Mwarabu | MK470294 |
| rpl23 | ribosomal protein L23 | Kenya_01_Local | MK470295 |
| rpl23 | ribosomal protein L23 | Kenya_02_Megana | MK470296 |
| rpl23 | ribosomal protein L23 | Kenya_03_Tereka | MK470297 |
| rpl23 | ribosomal protein L23 | Kenya_04_Megana | MK470298 |
| rpl23 | ribosomal protein L23 | Kenya_05_Megana | MK470299 |
| rpl23 | ribosomal protein L23 | Kenya_07_Local | MK470300 |
| rpl23 | ribosomal protein L23 | Kenya_10_Local | MK470301 |
| rpl23 | ribosomal protein L23 | Kenya_11_Kibandameno | MK470302 |
| rpl23 | ribosomal protein L23 | Kenya_12_Local | MK470303 |
| rpl23 | ribosomal protein L23 | Kenya_13_Kibandameno | MK470304 |
| rpl23 | ribosomal protein L23 | Kenya_14_Kibandameno | MK470305 |
| rpl23 | ribosomal protein L23 | Kenya_15_Kibandameno | MK470306 |
| rpl23 | ribosomal protein L23 | Mozambique_4_Ezalamalithi | MK470307 |
| rpl23 | ribosomal protein L23 | Mozambique_5_Calamidade | MK470308 |
| rpl23 | ribosomal protein L23 | Mozambique_8_Calamidade | MK470309 |
| rpl23 | ribosomal protein L23 | Mozambique_10_Bwana | MK470310 |
| rpl23 | ribosomal protein L23 | Mozambique_11_Fernando | MK470311 |
| rpl23 | ribosomal protein L23 | Mozambique_16_Bwana | MK470312 |
| rpl23 | ribosomal protein L23 | Mozambique_17_Mulaleia | MK470313 |
| rpl23 | ribosomal protein L23 | Mozambique_20_Cadri | MK470314 |
| rpl23 | ribosomal protein L23 | Mozambique_23_Mulaleia | MK470315 |
| rpl23 | ribosomal protein L23 | Mozambique_Manihot_glaziovii | MK470316 |
| rps7 | ribosomal protein S7 | Tanzania_Healthy | MK470317 |
| rps7 | ribosomal protein S7 | Tanzania_03_Rumara | MK470318 |
| rps7 | ribosomal protein S7 | Tanzania_04_Kibandameno | MK470319 |
| rps7 | ribosomal protein S7 | Tanzania_05_Mkunungu | MK470320 |
| rps7 | ribosomal protein S7 | Tanzania_06_Kibandameno | MK470321 |
| rps7 | ribosomal protein S7 | Tanzania_07_Unknown | MK470322 |
| rps7 | ribosomal protein S7 | Tanzania_08_Unknown | MK470323 |
| rps7 | ribosomal protein S7 | Tanzania_09_Mkangawandu | MK470324 |
| rps7 | ribosomal protein S7 | Tanzania_10_Kibembe | MK470325 |
| rps7 | ribosomal protein S7 | Tanzania_11_Kibembe | MK470326 |
| rps7 | ribosomal protein S7 | Tanzania_12_Mwarabu | MK470327 |
| rps7 | ribosomal protein S7 | Kenya_01_Local | MK470328 |
| rps7 | ribosomal protein S7 | Kenya_02_Megana | MK470329 |
| rps7 | ribosomal protein S7 | Kenya_03_Tereka | MK470330 |
| rps7 | ribosomal protein S7 | Kenya_04_Megana | MK470331 |
| rps7 | ribosomal protein S7 | Kenya_05_Megana | MK470332 |
| rps7 | ribosomal protein S7 | Kenya_07_Local | MK470333 |
| rps7 | ribosomal protein S7 | Kenya_10_Local | MK470334 |
| rps7 | ribosomal protein S7 | Kenya_11_Kibandameno | MK470335 |
| rps7 | ribosomal protein S7 | Kenya_12_Local | MK470336 |
| rps7 | ribosomal protein S7 | Kenya_13_Kibandameno | MK470337 |
| rps7 | ribosomal protein S7 | Kenya_14_Kibandameno | MK470338 |
| rps7 | ribosomal protein S7 | Kenya_15_Kibandameno | MK470339 |
| rps7 | ribosomal protein S7 | Mozambique_4_Ezalamalithi | MK470340 |
| rps7 | ribosomal protein S7 | Mozambique_5_Calamidade | MK470341 |
| rps7 | ribosomal protein S7 | Mozambique_8_Calamidade | MK470342 |
| rps7 | ribosomal protein S7 | Mozambique_10_Bwana | MK470343 |
| rps7 | ribosomal protein S7 | Mozambique_11_Fernando | MK470344 |
| rps7 | ribosomal protein S7 | Mozambique_16_Bwana | MK470345 |
| rps7 | ribosomal protein S7 | Mozambique_17_Mulaleia | MK470346 |
| rps7 | ribosomal protein S7 | Mozambique_20_Cadri | MK470347 |
| rps7 | ribosomal protein S7 | Mozambique_23_Mulaleia | MK470348 |
| rps7 | ribosomal protein S7 | Mozambique_Manihot_glaziovii | MK470349 |
| psaC | photosystem I subunit VII | Tanzania_Healthy | MK470350 |
| psaC | photosystem I subunit VII | Tanzania_03_Rumara | MK470351 |
| psaC | photosystem I subunit VII | Tanzania_04_Kibandameno | MK470352 |
| psaC | photosystem I subunit VII | Tanzania_05_Mkunungu | MK470353 |
| psaC | photosystem I subunit VII | Tanzania_06_Kibandameno | MK470354 |
| psaC | photosystem I subunit VII | Tanzania_07_Unknown | MK470355 |
| psaC | photosystem I subunit VII | Tanzania_08_Unknown | MK470356 |
| psaC | photosystem I subunit VII | Tanzania_09_Mkangawandu | MK470357 |
| psaC | photosystem I subunit VII | Tanzania_10_Kibembe | MK470358 |
| psaC | photosystem I subunit VII | Tanzania_11_Kibembe | MK470359 |
| psaC | photosystem I subunit VII | Tanzania_12_Mwarabu | MK470360 |
| psaC | photosystem I subunit VII | Kenya_01_Local | MK470361 |
| psaC | photosystem I subunit VII | Kenya_02_Megana | MK470362 |
| psaC | photosystem I subunit VII | Kenya_03_Tereka | MK470363 |
| psaC | photosystem I subunit VII | Kenya_04_Megana | MK470364 |
| psaC | photosystem I subunit VII | Kenya_05_Megana | MK470365 |
| psaC | photosystem I subunit VII | Kenya_07_Local | MK470366 |
| psaC | photosystem I subunit VII | Kenya_10_Local | MK470367 |
| psaC | photosystem I subunit VII | Kenya_11_Kibandameno | MK470368 |
| psaC | photosystem I subunit VII | Kenya_12_Local | MK470369 |
| psaC | photosystem I subunit VII | Kenya_13_Kibandameno | MK470370 |
| psaC | photosystem I subunit VII | Kenya_14_Kibandameno | MK470371 |
| psaC | photosystem I subunit VII | Kenya_15_Kibandameno | MK470372 |
| psaC | photosystem I subunit VII | Mozambique_4_Ezalamalithi | MK470373 |
| psaC | photosystem I subunit VII | Mozambique_5_Calamidade | MK470374 |
| psaC | photosystem I subunit VII | Mozambique_8_Calamidade | MK470375 |
| psaC | photosystem I subunit VII | Mozambique_10_Bwana | MK470376 |
| psaC | photosystem I subunit VII | Mozambique_11_Fernando | MK470377 |
| psaC | photosystem I subunit VII | Mozambique_16_Bwana | MK470378 |
| psaC | photosystem I subunit VII | Mozambique_17_Mulaleia | MK470379 |
| psaC | photosystem I subunit VII | Mozambique_20_Cadri | MK470380 |
| psaC | photosystem I subunit VII | Mozambique_23_Mulaleia | MK470381 |
| psaC | photosystem I subunit VII | Mozambique_Manihot_glaziovii | MK470382 |
| ndhE | NADH-plastoquinone oxidoreductase subunit 4L | Tanzania_Healthy | MK470383 |
| ndhE | NADH-plastoquinone oxidoreductase subunit 4L | Tanzania_03_Rumara | MK470384 |
| ndhE | NADH-plastoquinone oxidoreductase subunit 4L | Tanzania_04_Kibandameno | MK470385 |
| ndhE | NADH-plastoquinone oxidoreductase subunit 4L | Tanzania_05_Mkunungu | MK470386 |
| ndhE | NADH-plastoquinone oxidoreductase subunit 4L | Tanzania_06_Kibandameno | MK470387 |
| ndhE | NADH-plastoquinone oxidoreductase subunit 4L | Tanzania_07_Unknown | MK470388 |
| ndhE | NADH-plastoquinone oxidoreductase subunit 4L | Tanzania_08_Unknown | MK470389 |
| ndhE | NADH-plastoquinone oxidoreductase subunit 4L | Tanzania_09_Mkangawandu | MK470390 |
| ndhE | NADH-plastoquinone oxidoreductase subunit 4L | Tanzania_10_Kibembe | MK470391 |
| ndhE | NADH-plastoquinone oxidoreductase subunit 4L | Tanzania_11_Kibembe | MK470392 |
| ndhE | NADH-plastoquinone oxidoreductase subunit 4L | Tanzania_12_Mwarabu | MK470393 |
| ndhE | NADH-plastoquinone oxidoreductase subunit 4L | Kenya_01_Local | MK470394 |
| ndhE | NADH-plastoquinone oxidoreductase subunit 4L | Kenya_02_Megana | MK470395 |
| ndhE | NADH-plastoquinone oxidoreductase subunit 4L | Kenya_03_Tereka | MK470396 |
| ndhE | NADH-plastoquinone oxidoreductase subunit 4L | Kenya_04_Megana | MK470397 |
| ndhE | NADH-plastoquinone oxidoreductase subunit 4L | Kenya_05_Megana | MK470398 |
| ndhE | NADH-plastoquinone oxidoreductase subunit 4L | Kenya_07_Local | MK470399 |
| ndhE | NADH-plastoquinone oxidoreductase subunit 4L | Kenya_10_Local | MK470400 |
| ndhE | NADH-plastoquinone oxidoreductase subunit 4L | Kenya_11_Kibandameno | MK470401 |
| ndhE | NADH-plastoquinone oxidoreductase subunit 4L | Kenya_12_Local | MK470402 |
| ndhE | NADH-plastoquinone oxidoreductase subunit 4L | Kenya_13_Kibandameno | MK470403 |
| ndhE | NADH-plastoquinone oxidoreductase subunit 4L | Kenya_14_Kibandameno | MK470404 |
| ndhE | NADH-plastoquinone oxidoreductase subunit 4L | Kenya_15_Kibandameno | MK470405 |
| ndhE | NADH-plastoquinone oxidoreductase subunit 4L | Mozambique_4_Ezalamalithi | MK470406 |
| ndhE | NADH-plastoquinone oxidoreductase subunit 4L | Mozambique_5_Calamidade | MK470407 |
| ndhE | NADH-plastoquinone oxidoreductase subunit 4L | Mozambique_8_Calamidade | MK470408 |
| ndhE | NADH-plastoquinone oxidoreductase subunit 4L | Mozambique_10_Bwana | MK470409 |
| ndhE | NADH-plastoquinone oxidoreductase subunit 4L | Mozambique_11_Fernando | MK470410 |
| ndhE | NADH-plastoquinone oxidoreductase subunit 4L | Mozambique_16_Bwana | MK470411 |
| ndhE | NADH-plastoquinone oxidoreductase subunit 4L | Mozambique_17_Mulaleia | MK470412 |
| ndhE | NADH-plastoquinone oxidoreductase subunit 4L | Mozambique_20_Cadri | MK470413 |
| ndhE | NADH-plastoquinone oxidoreductase subunit 4L | Mozambique_23_Mulaleia | MK470414 |
| ndhE | NADH-plastoquinone oxidoreductase subunit 4L | Mozambique_Manihot_glaziovii | MK470415 |
| ndhG | NADH-plastoquinone oxidoreductase subunit 6 | Tanzania_Healthy | MK470416 |
| ndhG | NADH-plastoquinone oxidoreductase subunit 6 | Tanzania_03_Rumara | MK470417 |
| ndhG | NADH-plastoquinone oxidoreductase subunit 6 | Tanzania_04_Kibandameno | MK470418 |
| ndhG | NADH-plastoquinone oxidoreductase subunit 6 | Tanzania_05_Mkunungu | MK470419 |
| ndhG | NADH-plastoquinone oxidoreductase subunit 6 | Tanzania_06_Kibandameno | MK470420 |
| ndhG | NADH-plastoquinone oxidoreductase subunit 6 | Tanzania_07_Unknown | MK470421 |
| ndhG | NADH-plastoquinone oxidoreductase subunit 6 | Tanzania_08_Unknown | MK470422 |
| ndhG | NADH-plastoquinone oxidoreductase subunit 6 | Tanzania_09_Mkangawandu | MK470423 |
| ndhG | NADH-plastoquinone oxidoreductase subunit 6 | Tanzania_10_Kibembe | MK470424 |
| ndhG | NADH-plastoquinone oxidoreductase subunit 6 | Tanzania_11_Kibembe | MK470425 |
| ndhG | NADH-plastoquinone oxidoreductase subunit 6 | Tanzania_12_Mwarabu | MK470426 |
| ndhG | NADH-plastoquinone oxidoreductase subunit 6 | Kenya_01_Local | MK470427 |
| ndhG | NADH-plastoquinone oxidoreductase subunit 6 | Kenya_02_Megana | MK470428 |
| ndhG | NADH-plastoquinone oxidoreductase subunit 6 | Kenya_03_Tereka | MK470429 |
| ndhG | NADH-plastoquinone oxidoreductase subunit 6 | Kenya_04_Megana | MK470430 |
| ndhG | NADH-plastoquinone oxidoreductase subunit 6 | Kenya_05_Megana | MK470431 |
| ndhG | NADH-plastoquinone oxidoreductase subunit 6 | Kenya_07_Local | MK470432 |
| ndhG | NADH-plastoquinone oxidoreductase subunit 6 | Kenya_10_Local | MK470433 |
| ndhG | NADH-plastoquinone oxidoreductase subunit 6 | Kenya_11_Kibandameno | MK470434 |
| ndhG | NADH-plastoquinone oxidoreductase subunit 6 | Kenya_12_Local | MK470435 |
| ndhG | NADH-plastoquinone oxidoreductase subunit 6 | Kenya_13_Kibandameno | MK470436 |
| ndhG | NADH-plastoquinone oxidoreductase subunit 6 | Kenya_14_Kibandameno | MK470437 |
| ndhG | NADH-plastoquinone oxidoreductase subunit 6 | Kenya_15_Kibandameno | MK470438 |
| ndhG | NADH-plastoquinone oxidoreductase subunit 6 | Mozambique_4_Ezalamalithi | MK470439 |
| ndhG | NADH-plastoquinone oxidoreductase subunit 6 | Mozambique_5_Calamidade | MK470440 |
| ndhG | NADH-plastoquinone oxidoreductase subunit 6 | Mozambique_8_Calamidade | MK470441 |
| ndhG | NADH-plastoquinone oxidoreductase subunit 6 | Mozambique_10_Bwana | MK470442 |
| ndhG | NADH-plastoquinone oxidoreductase subunit 6 | Mozambique_11_Fernando | MK470443 |
| ndhG | NADH-plastoquinone oxidoreductase subunit 6 | Mozambique_16_Bwana | MK470444 |
| ndhG | NADH-plastoquinone oxidoreductase subunit 6 | Mozambique_17_Mulaleia | MK470445 |
| ndhG | NADH-plastoquinone oxidoreductase subunit 6 | Mozambique_20_Cadri | MK470446 |
| ndhG | NADH-plastoquinone oxidoreductase subunit 6 | Mozambique_23_Mulaleia | MK470447 |
| ndhG | NADH-plastoquinone oxidoreductase subunit 6 | Mozambique_Manihot_glaziovii | MK470448 |
| ndhI | NADH-plastoquinone oxidoreductase subunit I | Tanzania_Healthy | MK470449 |
| ndhI | NADH-plastoquinone oxidoreductase subunit I | Tanzania_03_Rumara | MK470450 |
| ndhI | NADH-plastoquinone oxidoreductase subunit I | Tanzania_04_Kibandameno | MK470451 |
| ndhI | NADH-plastoquinone oxidoreductase subunit I | Tanzania_05_Mkunungu | MK470452 |
| ndhI | NADH-plastoquinone oxidoreductase subunit I | Tanzania_06_Kibandameno | MK470453 |
| ndhI | NADH-plastoquinone oxidoreductase subunit I | Tanzania_07_Unknown | MK470454 |
| ndhI | NADH-plastoquinone oxidoreductase subunit I | Tanzania_08_Unknown | MK470455 |
| ndhI | NADH-plastoquinone oxidoreductase subunit I | Tanzania_09_Mkangawandu | MK470456 |
| ndhI | NADH-plastoquinone oxidoreductase subunit I | Tanzania_10_Kibembe | MK470457 |
| ndhI | NADH-plastoquinone oxidoreductase subunit I | Tanzania_11_Kibembe | MK470458 |
| ndhI | NADH-plastoquinone oxidoreductase subunit I | Tanzania_12_Mwarabu | MK470459 |
| ndhI | NADH-plastoquinone oxidoreductase subunit I | Kenya_01_Local | MK470460 |
| ndhI | NADH-plastoquinone oxidoreductase subunit I | Kenya_02_Megana | MK470461 |
| ndhI | NADH-plastoquinone oxidoreductase subunit I | Kenya_03_Tereka | MK470462 |
| ndhI | NADH-plastoquinone oxidoreductase subunit I | Kenya_04_Megana | MK470463 |
| ndhI | NADH-plastoquinone oxidoreductase subunit I | Kenya_05_Megana | MK470464 |
| ndhI | NADH-plastoquinone oxidoreductase subunit I | Kenya_07_Local | MK470465 |
| ndhI | NADH-plastoquinone oxidoreductase subunit I | Kenya_10_Local | MK470466 |
| ndhI | NADH-plastoquinone oxidoreductase subunit I | Kenya_11_Kibandameno | MK470467 |
| ndhI | NADH-plastoquinone oxidoreductase subunit I | Kenya_12_Local | MK470468 |
| ndhI | NADH-plastoquinone oxidoreductase subunit I | Kenya_13_Kibandameno | MK470469 |
| ndhI | NADH-plastoquinone oxidoreductase subunit I | Kenya_14_Kibandameno | MK470470 |
| ndhI | NADH-plastoquinone oxidoreductase subunit I | Kenya_15_Kibandameno | MK470471 |
| ndhI | NADH-plastoquinone oxidoreductase subunit I | Mozambique_4_Ezalamalithi | MK470472 |
| ndhI | NADH-plastoquinone oxidoreductase subunit I | Mozambique_5_Calamidade | MK470473 |
| ndhI | NADH-plastoquinone oxidoreductase subunit I | Mozambique_8_Calamidade | MK470474 |
| ndhI | NADH-plastoquinone oxidoreductase subunit I | Mozambique_10_Bwana | MK470475 |
| ndhI | NADH-plastoquinone oxidoreductase subunit I | Mozambique_11_Fernando | MK470476 |
| ndhI | NADH-plastoquinone oxidoreductase subunit I | Mozambique_16_Bwana | MK470477 |
| ndhI | NADH-plastoquinone oxidoreductase subunit I | Mozambique_17_Mulaleia | MK470478 |
| ndhI | NADH-plastoquinone oxidoreductase subunit I | Mozambique_20_Cadri | MK470479 |
| ndhI | NADH-plastoquinone oxidoreductase subunit I | Mozambique_23_Mulaleia | MK470480 |
| ndhI | NADH-plastoquinone oxidoreductase subunit I | Mozambique_Manihot_glaziovii | MK470481 |
| ndhA | NADH-plastoquinone oxidoreductase subunit 1 | Tanzania_Healthy | MK470482 |
| ndhA | NADH-plastoquinone oxidoreductase subunit 1 | Tanzania_03_Rumara | MK470483 |
| ndhA | NADH-plastoquinone oxidoreductase subunit 1 | Tanzania_04_Kibandameno | MK470484 |
| ndhA | NADH-plastoquinone oxidoreductase subunit 1 | Tanzania_05_Mkunungu | MK470485 |
| ndhA | NADH-plastoquinone oxidoreductase subunit 1 | Tanzania_06_Kibandameno | MK470486 |
| ndhA | NADH-plastoquinone oxidoreductase subunit 1 | Tanzania_07_Unknown | MK470487 |
| ndhA | NADH-plastoquinone oxidoreductase subunit 1 | Tanzania_08_Unknown | MK470488 |
| ndhA | NADH-plastoquinone oxidoreductase subunit 1 | Tanzania_09_Mkangawandu | MK470489 |
| ndhA | NADH-plastoquinone oxidoreductase subunit 1 | Tanzania_10_Kibembe | MK470490 |
| ndhA | NADH-plastoquinone oxidoreductase subunit 1 | Tanzania_11_Kibembe | MK470491 |
| ndhA | NADH-plastoquinone oxidoreductase subunit 1 | Tanzania_12_Mwarabu | MK470492 |
| ndhA | NADH-plastoquinone oxidoreductase subunit 1 | Kenya_01_Local | MK470493 |
| ndhA | NADH-plastoquinone oxidoreductase subunit 1 | Kenya_02_Megana | MK470494 |
| ndhA | NADH-plastoquinone oxidoreductase subunit 1 | Kenya_03_Tereka | MK470495 |
| ndhA | NADH-plastoquinone oxidoreductase subunit 1 | Kenya_04_Megana | MK470496 |
| ndhA | NADH-plastoquinone oxidoreductase subunit 1 | Kenya_05_Megana | MK470497 |
| ndhA | NADH-plastoquinone oxidoreductase subunit 1 | Kenya_07_Local | MK470498 |
| ndhA | NADH-plastoquinone oxidoreductase subunit 1 | Kenya_10_Local | MK470499 |
| ndhA | NADH-plastoquinone oxidoreductase subunit 1 | Kenya_11_Kibandameno | MK470500 |
| ndhA | NADH-plastoquinone oxidoreductase subunit 1 | Kenya_12_Local | MK470501 |
| ndhA | NADH-plastoquinone oxidoreductase subunit 1 | Kenya_13_Kibandameno | MK470502 |
| ndhA | NADH-plastoquinone oxidoreductase subunit 1 | Kenya_14_Kibandameno | MK470503 |
| ndhA | NADH-plastoquinone oxidoreductase subunit 1 | Kenya_15_Kibandameno | MK470504 |
| ndhA | NADH-plastoquinone oxidoreductase subunit 1 | Mozambique_4_Ezalamalithi | MK470505 |
| ndhA | NADH-plastoquinone oxidoreductase subunit 1 | Mozambique_5_Calamidade | MK470506 |
| ndhA | NADH-plastoquinone oxidoreductase subunit 1 | Mozambique_8_Calamidade | MK470507 |
| ndhA | NADH-plastoquinone oxidoreductase subunit 1 | Mozambique_10_Bwana | MK470508 |
| ndhA | NADH-plastoquinone oxidoreductase subunit 1 | Mozambique_11_Fernando | MK470509 |
| ndhA | NADH-plastoquinone oxidoreductase subunit 1 | Mozambique_16_Bwana | MK470510 |
| ndhA | NADH-plastoquinone oxidoreductase subunit 1 | Mozambique_17_Mulaleia | MK470511 |
| ndhA | NADH-plastoquinone oxidoreductase subunit 1 | Mozambique_20_Cadri | MK470512 |
| ndhA | NADH-plastoquinone oxidoreductase subunit 1 | Mozambique_23_Mulaleia | MK470513 |
| ndhA | NADH-plastoquinone oxidoreductase subunit 1 | Mozambique_Manihot_glaziovii | MK470514 |
| ndhH | NADH-plastoquinone oxidoreductase subunit 7 | Tanzania_Healthy | MK470515 |
| ndhH | NADH-plastoquinone oxidoreductase subunit 7 | Tanzania_03_Rumara | MK470516 |
| ndhH | NADH-plastoquinone oxidoreductase subunit 7 | Tanzania_04_Kibandameno | MK470517 |
| ndhH | NADH-plastoquinone oxidoreductase subunit 7 | Tanzania_05_Mkunungu | MK470518 |
| ndhH | NADH-plastoquinone oxidoreductase subunit 7 | Tanzania_06_Kibandameno | MK470519 |
| ndhH | NADH-plastoquinone oxidoreductase subunit 7 | Tanzania_07_Unknown | MK470520 |
| ndhH | NADH-plastoquinone oxidoreductase subunit 7 | Tanzania_08_Unknown | MK470521 |
| ndhH | NADH-plastoquinone oxidoreductase subunit 7 | Tanzania_09_Mkangawandu | MK470522 |
| ndhH | NADH-plastoquinone oxidoreductase subunit 7 | Tanzania_10_Kibembe | MK470523 |
| ndhH | NADH-plastoquinone oxidoreductase subunit 7 | Tanzania_11_Kibembe | MK470524 |
| ndhH | NADH-plastoquinone oxidoreductase subunit 7 | Tanzania_12_Mwarabu | MK470525 |
| ndhH | NADH-plastoquinone oxidoreductase subunit 7 | Kenya_01_Local | MK470526 |
| ndhH | NADH-plastoquinone oxidoreductase subunit 7 | Kenya_02_Megana | MK470527 |
| ndhH | NADH-plastoquinone oxidoreductase subunit 7 | Kenya_03_Tereka | MK470528 |
| ndhH | NADH-plastoquinone oxidoreductase subunit 7 | Kenya_04_Megana | MK470529 |
| ndhH | NADH-plastoquinone oxidoreductase subunit 7 | Kenya_05_Megana | MK470530 |
| ndhH | NADH-plastoquinone oxidoreductase subunit 7 | Kenya_07_Local | MK470531 |
| ndhH | NADH-plastoquinone oxidoreductase subunit 7 | Kenya_10_Local | MK470532 |
| ndhH | NADH-plastoquinone oxidoreductase subunit 7 | Kenya_11_Kibandameno | MK470533 |
| ndhH | NADH-plastoquinone oxidoreductase subunit 7 | Kenya_12_Local | MK470534 |
| ndhH | NADH-plastoquinone oxidoreductase subunit 7 | Kenya_13_Kibandameno | MK470535 |
| ndhH | NADH-plastoquinone oxidoreductase subunit 7 | Kenya_14_Kibandameno | MK470536 |
| ndhH | NADH-plastoquinone oxidoreductase subunit 7 | Kenya_15_Kibandameno | MK470537 |
| ndhH | NADH-plastoquinone oxidoreductase subunit 7 | Mozambique_4_Ezalamalithi | MK470538 |
| ndhH | NADH-plastoquinone oxidoreductase subunit 7 | Mozambique_5_Calamidade | MK470539 |
| ndhH | NADH-plastoquinone oxidoreductase subunit 7 |  |  |
| ndhH | NADH-plastoquinone oxidoreductase subunit 7 | Mozambique_8_Calamidade | MK470540 |
| ndhH | NADH-plastoquinone oxidoreductase subunit 7 | Mozambique_10_Bwana | MK470541 |
| ndhH | NADH-plastoquinone oxidoreductase subunit 7 | Mozambique_11_Fernando | MK470542 |
| ndhH | NADH-plastoquinone oxidoreductase subunit 7 | Mozambique_16_Bwana | MK470543 |
| ndhH | NADH-plastoquinone oxidoreductase subunit 7 | Mozambique_17_Mulaleia | MK470544 |
| ndhH | NADH-plastoquinone oxidoreductase subunit 7 | Mozambique_20_Cadri | MK470545 |
| ndhH | NADH-plastoquinone oxidoreductase subunit 7 | Mozambique_23_Mulaleia | MK470546 |
| ndhH | NADH-plastoquinone oxidoreductase subunit 7 | Mozambique_Manihot_glaziovii | MK470547 |
